# miR-198 targets *TOPORS*: implications for oral squamous cell carcinoma pathogenesis

**DOI:** 10.3389/fonc.2024.1485802

**Published:** 2024-12-04

**Authors:** Pankhuri Kaushik, Radha Mishra, Champaka Gopal, Arun Kumar

**Affiliations:** ^1^ Department of Developmental Biology and Genetics, Indian Institute of Science, Bangalore, India; ^2^ Department of Pathology, Kidwai Memorial Institute of Oncology, Bangalore, India

**Keywords:** MiR-198, MIR198 promoter, methylation, tumor suppressor, TOPORS, OSCC

## Abstract

**Background:**

miRNAs play a critical role in the progression of various diseases, including oral squamous cell carcinoma (OSCC), which represents a major health concern and is one of the leading causes for new cancer cases worldwide. The miRNA dysregulation causes havoc and could be attributed to various factors, with epigenetic silencing of tumor suppressor genes being a major contributor to tumorigenesis. In this study, we have explored the tumor suppressive role of miR-198 in OSCC.

**Methods:**

The tumor suppressive effect of miR-198 is established using miRNA analysis in OSCC cell lines, patient samples and xenograft nude mice model. The relationship between the miR-198 and *TOPORS* is explored using bioinformatics analyses, qRT-PCR, dual-luciferase reporter assay, Western blotting and cancer hall marks assays. The hypermethylation of the *MIR198* promoter is confirmed using bisulfite sequencing PCR.

**Results:**

We have found miR-198 to be upregulated in OSCC cells treated with 5-Azacytidine, a known DNA methyltransferase inhibitor. Upregulation of miR-198 in 5-Azacytidine treated OSCC cells appears to be due to methylation of the *MIR198* promoter. Using bioinformatics analysis and dual-luciferase reporter assay, we have identified *TOPORS* (TOP1 binding arginine/serine rich protein, E3 ubiquitin ligase) as a novel gene target for miR-198. miR-198-mediated repression of TOPORS decreases cell proliferation and anchorage-independent growth and enhances apoptosis of OSCC cells, which is dependent on the presence of the 3′UTR in *TOPORS*. An inverse correlation between the expression levels of miR-198 and *TOPORS* is observed in OSCC patient samples, highlighting the biological relevance of their interaction. Delivery of a synthetic miR-198 mimic to OSCC cells results in a significant decrease in xenograft size in nude mice, potentiating its use in therapeutics.

**Conclusions:**

These results suggest that miR-198 is epigenetically silenced in OSCC, which promotes tumor growth, in part, by upregulating the levels of *TOPORS*.

## 1 Introduction

Oral squamous cell carcinoma (OSCC) is responsible for incidence of 3,77,713 new cases and 1,77,757 deaths in both sexes worldwide (1). The situation is worse for India as it ranks first in the rate of incidence of lip and oral cavity cancers across countries worldwide (2). Despite easy access of tumors in the oral cavity, patients present with advanced tumors due to lack of awareness, leading to late detection. In the case of early diagnosis, the 5-year survival rate can be more than 80%, but most patients are diagnosed at advanced stages with low (39%) 5-year survival rate (3). Drawbacks in the current treatment regimens, despite several technological advancements, are majorly responsible for the lack of improvement in the 5-year survival rates during the past few decades (4). It thus creates an urgent need to identify novel therapeutic modalities for OSCC, which not only improve prognosis but also diagnosis and patient survival. Increasing evidence reveals a new class of small non-coding RNAs (ncRNAs), the miRNAs as therapeutic targets in numerous diseases including cancers (5). Since their discovery, the role of miRNA dysregulation in disease progression has widely been studied which highlights the potential that miRNAs hold in the treatment of these diseases. The understanding of such miRNA-gene target axis could open new avenues for personalized cancer therapy.

miRNAs post-transcriptionally regulate the expression of their target genes by either inducing degradation or by inhibiting translation (5). Reports from the last few decades have underlined the pivotal role miRNAs play in the maintenance of homeostasis by regulating several cellular processes (6, 7). Their abnormal expression has been linked to development of various diseases including OSCC (8, 9). miRNAs such as miR-137, miR-126a, miR-99a, miR-375, miR-6741-3p and miR-617 are reported to be downregulated and potentially tumor suppressive, whereas miR-21, miR-27a, miR-181b, miR-131, miR-155 and miR-130a are reported to be upregulated and oncogenic in OSCC (10–14). Restoration of tumor suppressive miRNAs and knockdown of oncogenic miRNAs can be used as novel therapy for cancer treatment. Interestingly in cancer, miRNAs expression profiles present with more miRNA subsets with lower expression levels compared with the normal tissue, indicative of tumor suppressive functions (15). Also, miRNA genes are located in the cancer-associated genomic region, which are frequently deleted in cancers (16). The exact mechanisms for regulation of miRNA expression remain less understood, with epigenetic silencing, defective miRNA biogenesis machinery, and genetic changes being some of the players (17).

Epigenetic modifications seem to be the major mechanism responsible for miRNA expression disruptions in cancer (18). Silencing or downregulation of tumor suppressor miRNAs by aberrant methylation of CpG sites located in their promoters is demonstrated by several studies and found to be associated with prognosis of the disease and response to therapy (19–22). Further, ~50% miRNAs are found to be embedded in CpG islands (23). Studies demonstrate that ectopic unmasking of epigenetically silenced miR-127 and miR-124a restored their expressions in bladder cancer and colorectal cancer cells respectively, highlighting a method for identification of epigenetically silenced tumor suppressor miRNAs (24, 25). Kozaki et al. (26) have identified for the first time miR-137 and miR-193a as epigenetically silenced miRs due to DNA methylation in OSCC.

A previous study from our laboratory analyzed the miRNA microarray expression profile of 5-Azacytidine treated cells from an OSCC cell line SCC131 to identify novel tumor suppressor miRNAs. The analysis yielded 50 upregulated microRNAs, including miR-198 (13). Various oncogenes and signaling pathways have been identified as targets for miR-198, elucidating its role as tumor suppressor in prostate cancer, gastric cancer, lung adenocarcinoma, hepatocellular carcinoma, breast cancer, colorectal cancer, head and neck squamous cell carcinoma, thyroid cancer, osteosarcoma and pancreatic cancer (27). Some of the targets of miR-198 include well known oncogenes like livin (*BIRC7*), mindbomb E3 ubiquitin protein ligase 1 (*MIB1*), fibroblast growth factor receptor 1 (*FGFR1*), MET Proto-Oncogene, receptor tyrosine kinase (*MET*), ADAM metallopeptidase domain 28 (*ADAM 28*), and fucosyl transferase 8 (*FUT8*). The dysregulation of miR-198 in these diseases indicates its key role in the maintenance of normal tissue homeostasis and highlights the potential of miR-198 replacement therapy for cancer treatment. Despite being widely studied in several diseased conditions including cancers (27), not much is known about the role of miR-198 in OSCC, except with a single report by Kang et al. (4), where they have elucidated the role of miR-198 in OSCC pathogenesis via its gene target *CDK4*.

Here, we report the role of miR-198 as a tumor suppressor in OSCC and its novel target *TOPORS*. We elucidated the mechanism for upregulation of miR-198 following 5-Azacytidine treatment of cells from an OSCC cell line SCC131. Further, we have also investigated if the restoration of miR-198 level by a synthetic miR-198 mimic can have an antitumor effect *in vivo*.

## 2 Materials and methods

### 2.1 OSCC cell lines and patient samples

The cells from human OSCC cell lines, UPCI: SCC084 and UPCI: SCC131, were grown in DMEM supplemented with 10% fetal bovine serum and 1x antibiotic/antimycotic solution (Sigma-Aldrich, St. Louis, MO) in a humidified chamber with 5% CO_2_ at 37°C. These cell lines were a kind gift from Prof. Susanne Gollin, University of Pittsburgh, Pittsburgh, PA (28). These cell lines were generated following Institutional Review Board guidelines from consenting patients undergoing surgery for squamous cell carcinoma of the oral cavity at the University of Pittsburgh Medical Centre (28). SCC131 cells were derived from a T2N2bM0 lesion on the floor of the mouth of a 73-year-old Caucasian male, and SCC084 cells were derived from a T2N2M0 lesion of the retromolar trigone of a 52-year-old Caucasian male (28). A total of 39 matched OSCC tumor and their adjacent normal oral tissues from the patients were obtained at the Kidwai Memorial Institute of Oncology (KMIO), Bangalore from July 13, 2018 to November 14, 2018. The samples were obtained as surgically resected tissues from oral cancerous lesions and adjacent normal tissues (taken from the farthest margin of surgical resection) in the RNALater (Sigma-Aldrich, St. Louis, MO) and transferred to -80°C until further use (13, 14). The clinicopathological information for the 39 patients is given in **Supplementary Tables S1**, **S2**. Tumors were classified according to tumor, node, and metastasis criteria (29).

### 2.2 Transient transfections and reporter assays

For the overexpression studies, SCC131 or SCC084 cells were seeded at 2×10^6^ cells/well in a 6-well plate and transfected with an appropriate construct or a combination of different constructs using Lipofectamine 2000 (Thermo Fisher Scientific, Waltham, MA) according to the manufacturer’s instructions. For the dual-luciferase reporter assay, 5 × 10^4^ cells/well were transfected in 24-well plates with different constructs. The dual-luciferase reporter assays were performed post 48 h of transfection using the Dual-Luciferase Reporter Assay System (Promega, Madison, WI) according to the manufacturer’s protocol. The pRL-TK control vector (Promega, Madison, WI) was co-transfected in the cells for normalizing the transfection efficiency (10).

### 2.3 Total RNA isolation and cDNA preparation

Total RNA from tissues and cells was isolated using TRI-Reagent (Sigma-Aldrich, St. Louis, MO) and quantitated using a NanoDrop 1000 spectrophotometer (Thermo Fischer Scientific, Waltham, MA). The first-strand cDNA was synthesized from 2 µg of total RNA using a Verso cDNA Synthesis Kit (Thermo Fischer Scientific, Waltham, MA).

### 2.4 qRT-PCR analysis

The expression level of miR-198 was determined by qRT-PCR using the miR-Q technique by Sharbati-Tehrani et al. (30). The details of the primers are given in **Supplementary Table S3**. The RT-qPCR analysis was performed using the DyNAmo ColorFlash SYBR Green qPCR Kit in StepOne Plus Real-Time PCR and QuantStudio 3 PCR Systems (Thermo Fischer Scientific, Waltham, MA). *GAPDH* and *5S rRNA* were used as normalizing controls (13). The following equation ΔCt_gene_ = Ct_gene_-Ct_normalizing control_, was used to calculate the fold change. Ct represents cycle threshold value, and ΔCt represents the gene expression normalized to *GAPDH* or *5S rRNA*.

### 2.5 Plasmid constructs

The miR-198 (pmiR-198) construct was generated in pcDNA3-EGFP, using human genomic DNA as a template and gene specific primers (**Supplementary Table S4**) by a standard laboratory method. The *TOPORS*-ORF construct (p*TOPORS*) in the pcDNA 3.1(+) vector (Invitrogen, Waltham, MA, USA) was obtained from the GenScript (cat # OHu18284C, ORF size- 3138 bp, Piscataway, NJ). For the validation of a direct interaction between miR-198 and the 3’UTR of *TOPORS* by the dual-luciferase reporter assay, we cloned the 3’UTR of *TOPORS* in a sense orientation in the pMIR-REPORT miRNA Expression Reporter Vector System (Thermo Fischer Scientific, Waltham, MA) downstream to the luciferase (Luc) gene using human genomic DNA as a template. Briefly, the *TOPORS* sequences were obtained from the UCSC Genome browser (https://genome.ucsc.edu/) and the 769 bp long fragment was amplified from human genomic DNA using specific primers (**Supplementary Table S4**). The fragment was first cloned in a cloning vector pBluescript II KS (+) [pBSKS (+)] and the construct was named as pBSKS-TOPORS-3’UTR-S. From this vector, we excised the 3’UTR fragment with *Pme* I and *Mlu* I and ligated it to the pMIR-REPORT vector, and this construct was named as pMIR-REPORT-*TOPORS*-3’UTR-S. To validate the sequence specificity of the interaction between miR-198 and the *TOPORS* 3’UTR, the target site for miR-198 present in the 3’UTR of *TOPORS* was abrogated by site-directed mutagenesis (31). For this, the construct pBSKS-*TOPORS*-3’UTR-M was generated using specific primers (**Supplementary Table S4**) and pBSKS-*TOPORS*-3’UTR-S (carrying the *TOPORS* 3’UTR in a sense orientation) as the template. The mutated *TOPORS* 3’UTR was excised from this construct with *Pme I* and *Mlu I* and ligated in the pMIR-REPORT vector (also digested with the same enzymes) to generate the pMIR-REPORT-*TOPORS*-3’UTR-M construct. To determine if the 3’UTR of *TOPORS* is responsible for miR-198-mediated regulation of *TOPORS*, the p*TOPORS*-3′UTR-S, and p*TOPORS*-3′UTR-M constructs were generated by cloning the relevant 3′UTR sequences downstream to the *TOPORS*-ORF at *EcoR* V and *Not* I restriction sites in the p*TOPORS* construct (**Supplementary Table S4**). All constructs used in the study were validated by restriction enzyme digestion and Sanger sequencing on a 3730xl DNA analyser (Thermo Fisher Scientific, Waltham, MA, USA).

### 2.6 Western hybridization

Protein lysates from cells were prepared using the CelLytic M Cell Lysis Reagent (Sigma-Aldrich, St. Louis, MO). The proteins were then resolved on an SDS-PAGE and then transferred on a PVDF membrane (Pall Corp., Port Washington, NY). The membrane was blocked using 5% fat-free milk powder in 1× PBST (1xX Phosphate-Buffered Saline, 0.1% Tween 20 Detergent), and the signal was visualized using an appropriate antibody and the Immobilon Western Chemiluminescent HRP substrate (Milipore, Billerica, MA). The following antibodies were used: anti-rabbit-TOPORS (1:1,000 dilution; cat# ab86383, Abcam, Cambridge, CAMBS, UK), anti-mouse β-actin (1:10,000 dilution; cat# A5441, Sigma-Aldrich, St. Louis, MO), p53 wild-type (1: 1,000 dilution; cat# AH00152, Thermo Fisher Scientific, Waltham, MA), p21 Waf1/Cip1 (1:2,500 dilution; cat#2946, CST, Danvers, MA) and secondary HRP-conjugated goat anti-rabbit/HRP-conjugated goat anti-mouse (1.5:5,000 dilution; Bangalore Genei, Bangalore, India) antibodies.

### 2.7 Cell proliferation assay

To analyze the rate of cell proliferation, a trypan blue dye exclusion assay was employed (13, 32). Briefly, 2x10^6^ cells were seeded in 6-well plates. After 16 hr, cells were transfected with the desired construct(s) as described above. Post 24 h of transfection, they were trypsinized, counted and reseeded at the density of 30,000 cells/well in triplicates in four 24-well plates (one plate for each day; total 4 days). This is day 0. Starting from day 1 (24 h after reseeding), cells from each well were trypsinized. The cells were then diluted 1:10 with 0.4% trypan blue dye (Sigma-Aldrich, St. Louis, MO) prepared in 1XPBS, allowed to stain for 10 min at room temperature and counted using a hemacytometer. This was done for all 4 days. The number of viable cells/mL was calculated using the following formula: number of viable cells/mL = the average count per square (unstained/live cells) x dilution factor x 10^4^ (13).

### 2.8 Detection of caspase-3 activation

The CaspGLOW Fluorescein Active Caspase-3 Staining Kit (Biovision, Mountain View, CA) was used to analyze the rate of apoptosis in cells transfected with the appropriate constructs (10). After transfection, FITC-DEVD label was added to cells, and the rest of the steps were followed as per the manufacturer’s protocol. The fluorescence percentage was measured, using FACSCalibur Flow cytometer (BD Biosciences, New Jersey, NJ), using the FL-1 channel and the BD Accuri C6 software.

### 2.9 Soft agar colony forming assay

The anchorage-independent growth is one of the hallmarks of cancer, where the cancer cells have the ability to grow independently on a solid surface and was assessed by the number of colonies formed in soft agar (10, 13, 14). After transfection of cells with different constructs, 6,000 cells were plated in 1 ml of 0.35% Difco Noble Agar (Difco, Mumbai, India) diluted with DMEM culture media in a 35 mm dish. After 20-25 days, colonies were stained with 0.01% crystal violet (Sigma-Aldrich, St. Louis, MO), counted and imaged using a Leica Inverted Microscope DMi1 (Leica Microsystems, Wetzlar, Germany).

### 2.10 Identification of the *MIR198* promoter

Although Duan et al. (33) have earlier identified an independent promoter for miR-198, a thorough analysis of the primers used by them for the promoter sequence revealed the presence of the promoter in another gene, homogentisate 1,2-dioxygenase (*HGD*; NC_000003.12-120628172-120682239, complement) and not in the *FSTL1* gene (NC_000003.12-120392293-120450992, complement), which harbours the *MIR198* gene. Therefore, we used the following databases to predict the putative *MIR198* gene promoter: DBTSS (http://dbtss.hgc.jp/), MatInspector (https://www.genomatix.de) and Switchgear (https://switchgeargenomics.com). The predicted DNA sequence for the *MIR198* promoter was then cloned upstream to the luciferase ORF in the pGL3-Basic vector (Promega, Madison, OH), using a standard laboratory method and specific primers (**Supplementary Table S5**). The resulting promoter construct, pMIR198-prom was then used for the promoter reporter assay using the Dual-Luciferase Reporter Assay system (Promega, Madison, WI, USA), according to manufacturer’s instructions.

### 2.11 Genomic bisulfite sequencing

For demethylation experiments, SCC131 cells were grown in T-75 flasks till 80% confluency and treated with a freshly prepared 5-Azacytidine at 5 μM final concentration (Sigma-Aldrich, St. Lous, MO) for 5 days with the drug and the medium being replaced every 24 h. DMSO (Sigma-Aldrich, St. Lous, MO) was used as a vehicle control. Following treatment, cells were harvested, and the genomic DNA was extracted using a Wizard Genomic DNA Purification Kit (Promega, Madison, WI, USA), according to the manufacturer’s instructions. An aliquot of 2 µg of total genomic DNA from each treatment was treated with sodium bisulphite (10, 14). Using sodium bisulphite treated genomic DNA from control (DMSO) and 5-Azacytidine treated SCC131 cells as templates, the *MIR198* promoter-specific fragment was amplified (**Supplementary Table S6**) using methylation specific primers and cloned in the pTZ57R TA cloning vector (Thermo Fisher Scientific, Waltham, MA). Ten random TA clones were selected for each treatment and Sanger sequenced as described above. Methylation-specific primers were designed using the MethPrimer database (http://www.urogene.org/methprimer/).

### 2.12 *In silico*analysis of gene targets for miR-198

A consensus approach was employed to predict the gene targets for miR-198 using following mRNA target prediction algorithms like miRDB (http://mirdb.org), TargetScanHuman (https://www.targetscan.org/vert_80/), PicTar (https://pictar.mdc-berlin.de/cgi-bin/PicTar_vertebrate.cgi) and CoMeTa (https://cometa.tigem.it/) (**Supplementary Table S7**).

### 2.13 *In vivo* study in nude mice

To analyze the effect of miR-198-mediated targeting of *TOPORS* on tumor growth, 2×10^6^ SCC131 cells were transfected with 900 nM of a synthetic miR-198 mimic or 900 nM of a mock control (scrambled oligos) separately. After 24 h of transfection, cells from both groups were suspended separately in 150 μl of incomplete DMEM and then injected into the right flank of a female BALB/c athymic 6-week-old nude mouse subcutaneously (10, 13, 14). Tumors were allowed to grow in animals of the two experimental sets, and tumor volumes were measured using a Vernier’s calliper every alternate day till the termination of the experiment. At the end of the study, animals were euthanized in CO_2_ atmosphere under sterile conditions by cervical dislocation. Tumor volumes were calculated using the formula: V = L×W^2^×0.5, where L and W represent the length and width of the tumor, respectively (10, 13, 14). The animals were photographed, and the tumor xenografts were harvested, photographed and weighed at the end of the study by trained personnel. Mice were maintained on a 12:12 h light/dark cycle in proper cages with sufficient food and water. Efforts were taken to alleviate suffering of the animals, and they were handled and treated only by trained personnel. Animals were consistently monitored for general health and behaviour. miRIDIAN microRNA hsa-miR-198 Hairpin mimic (cat# C-300532-05-0020) and miRIDIAN microRNA Negative Control #1/Mock (cat# CN-001000-01-XX) were purchased from Dharmacon (Lafayette, CO).

### 2.14 Statistical analysis

The statistical significance of the comparison between any two experimental data sets was calculated using the student’s t test in the GraphPad Prism 8 software (Boston, MA, USA). The statistical significance of comparisons between multiple data sets was calculated by ANOVA (one-way analysis of variance). Comparisons in data sets were considered significant when p-values were ≤0.05 (*), <0.01 (**), <0.001 (***), <0.0001 (****) or non-significant (ns) when the p-value was >0.05.

## 3 Results

### 3.1 Validation of 5-Azacytidine induced upregulation of miR-198 and its anti-proliferative role in OSCC cells

**Figure 1:**
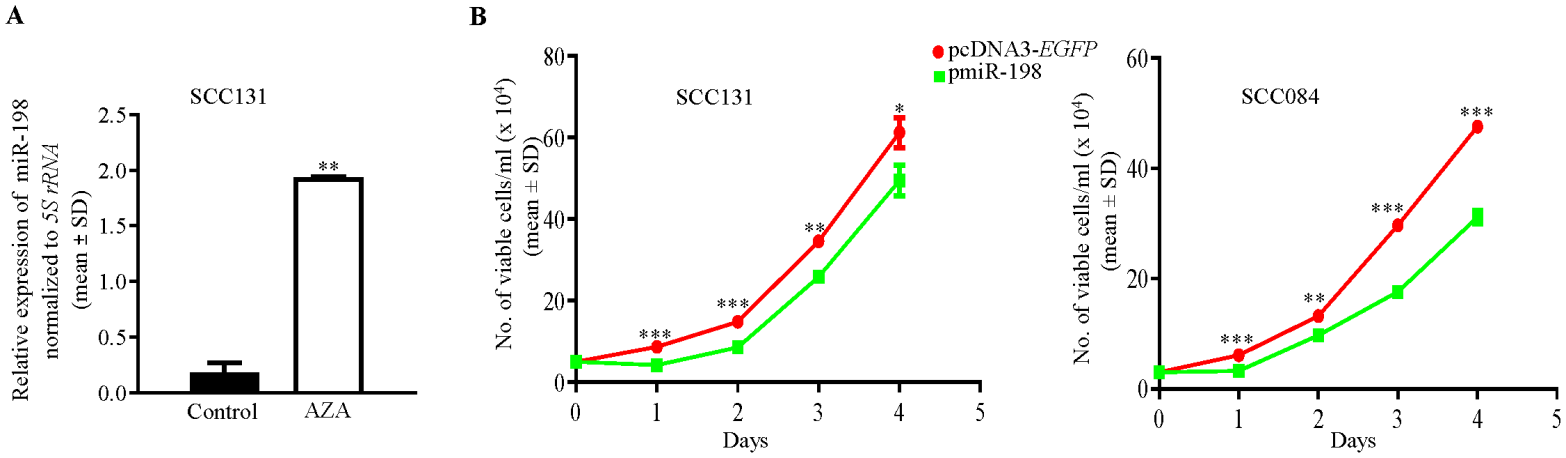
Validation of upregulation of miR-198 in 5-Azacytidine treated cells and its antiproliferative role. **(A)** 5-Azacytidine upregulates miR-198 in SCC131 cells compared to those treated with the control (DMSO). **(B)** MiR-198 ectopic overexpression suppresses the proliferation of SCC131 and SCC084 cells. The trypan blue dye exclusion assay was used to analyze transient overexpression of miR-198 on cell proliferation, using the pmiR-198 construct and cloning vector pcDNA3-EGFP. Note, a significant reduction in proliferation of both SCC131 and SCC084 cells transfected with the pmiR-198 construct compared to those transfected with the vector control. Each data point is an average of 3 biological replicates.

We first validated the upregulation of miR-198 in 5-Azacytidine treated SCC131 cells, using RT6-198 and short-miR-198 primers specific to miR-198 (**Supplementary Table S3**). A significant upregulation in the miR-198 level was observed in 5-Azacytidine treated cells compared with the vehicle (DMSO) treated cells, thus validating the human miRNA microarray data (**Figure 1A**).

DNA hypermethylation is one of the key players for the reduced expression of tumor suppressor miRNAs in cancer. As 5-Azacytidine is a global hypomethylating agent which can reactivate the transcription of tumor suppressor miRNAs, and miR-198 was observed to be upregulated post 5-Azacytidine treatment of SCC131 cells, we hypothesized that miR-198 could be a tumor suppressor miRNA. We, therefore, wanted to explore if overexpression of miR-198 in OSCC cells has an anti-proliferative role in OSCC cells. We then transfected SCC131 and SCC084 cells with pcDNA3-EGFP (vector control) and pmiR-198 separately and performed the trypan blue dye exclusion cell counting assay. The results showed that miR-198 significantly reduced the rate of proliferation of OSCC cells compared with those transfected the vector control, suggesting that it negatively regulates cell proliferation and functions as a tumor suppressor in OSCC cells (**Figure 1B**).

### 3.2 Mechanism for miR-198 upregulation following 5-Azacytidine treatment of SCC131 cells

To understand the underlying mechanism for the upregulation of miR-198 in 5-Azacytidine treated SCC131 cells, we hypothesized that the demethylation of the *MIR198* promoter can cause upregulation of miR-198 levels. Since the promoter of miR-198 is unknown, we first retrieved the putative *MIR198* promoter sequence from the DBTSS, Switchgear and Matinspector databases (**Supplementary Figure S1A**). The 839 bp sequence (**Supplementary Figure S1A**) was cloned in the pGL3-Basic vector. The *MIR198* promoter construct (pMIR198-prom; **Supplementary Figure S1B**) along with the positive control pGL3-Control and the negative control pGL3-Basic were then transfected separately in SCC131 and SCC084 cells and the dual-luciferase reporter assay was performed. The results showed a significant promoter activity for the *MIR198* promoter construct in both SCC131 and SCC084 cells compared with the pGL3-Basic vector (**Supplementary Figure S1C**), suggesting that the putative *MIR198* promoter sequence indeed represents an independent promoter for the *MIR198* gene.

**Figure 2:**
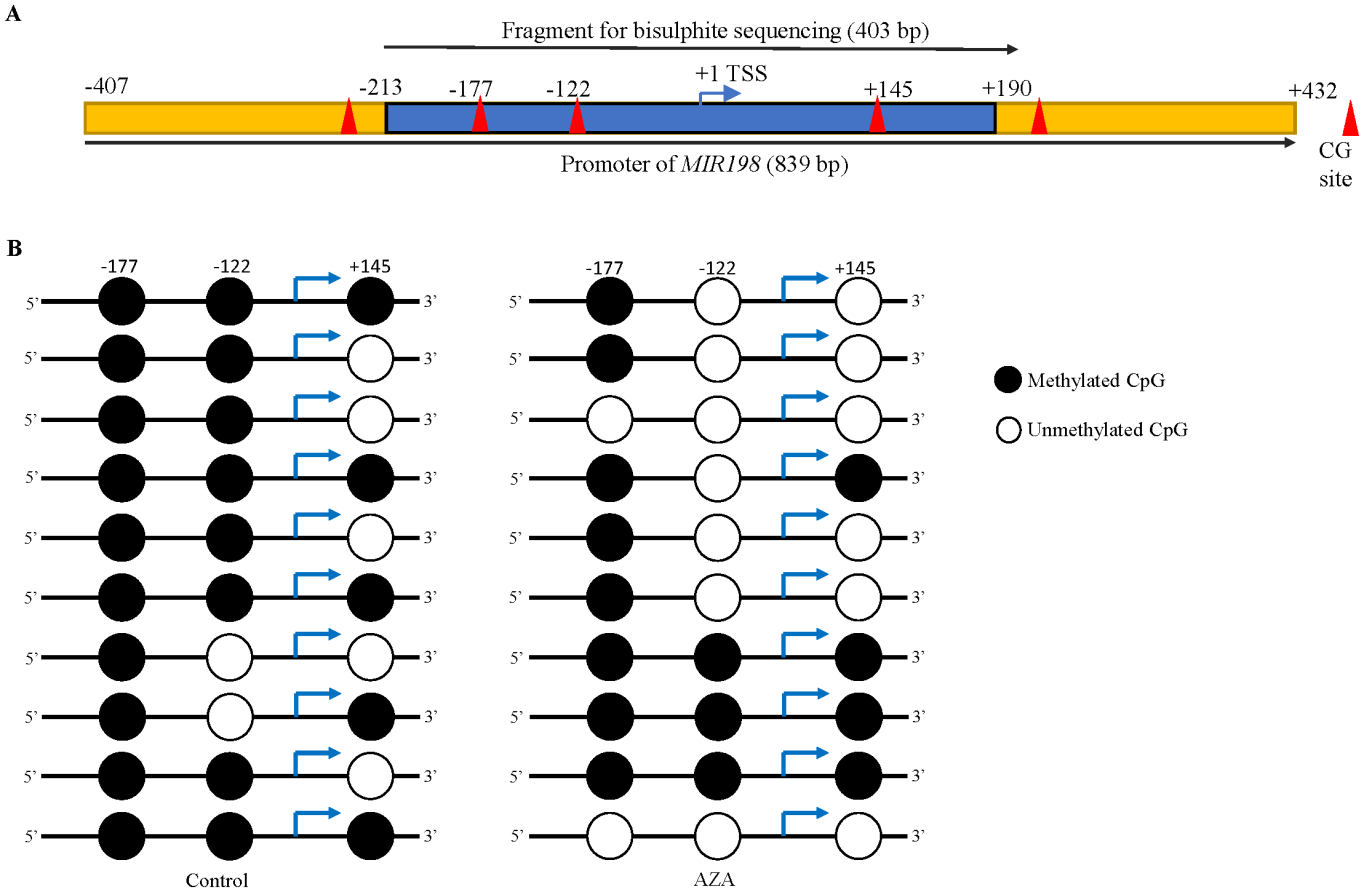
Demethylation of the *MIR198* promoter following 5-Azacytidine treatment of SCC131 cells. **(A)** The schematic diagram of the *MIR198* promoter region. The blue portion was analyzed for bisulphite sequencing and the cloned sequence contains 3 CpG sites, highlighted as red triangles. **(B)** Methylation status of 3 CpG sites in the *MIR198* promoter fragment from control (left panel) and 5-Azacytidine treated SCC131 cells (right panel). Each row represents methylation status of CpGs from a single TA clone.

After characterizing the *MIR198* promoter, we analysed the methylation status of the *MIR198* promoter, using bisulphite sequencing PCR (BSP). We have designed BSP specific primers (**Supplementary Table S6**) from nucleotide positions -213 to +190, which harbours 3 CpG sites (**Figure 2A**). Using sodium bisulphite treated genomic DNA from control and 5-Azacytidine treated SCC131 cells as templates, we amplified the *MIR198* promoter-specific fragment with BSP-specific primers (**Supplementary Table S6**), cloned them in a TA cloning vector, and Sanger sequenced 10 random clones for each of the control and 5-Azacytidine treated SCC131 cells. The results showed that the percentage of methylation at the *MIR198* promoter region reduced from 76.66% in the control-treated SCC131 cells to 50% in 5-Azacytidine treated cells (**Figure 2B**), suggesting that demethylation of the *MIR198* promoter is responsible for upregulation of miR-198 following 5-Azacytidine treatment of SCC131 cells.

### 3.3 Identification of a gene target for miR-198

MiRNAs are known to regulate gene expression by binding to their cognate mRNA targets. As we have observed miR-198 to have an anti-proliferative role in OSCC cells, we wanted to elucidate its target gene(s) and the mechanism through which it exerts its effect. To this end, we have used a consensus approach by employing four different mRNA target prediction algorithms (e.g., TargetScanHuman, miRDB, PicTar and CoMeTa) to identify gene target(s) of miR-198. We found *TOPORS* (topoisomerase I binding, arginine/serine-rich, E3 ubiquitin protein ligase), *NRIP1* (nuclear receptor interacting protein 1), *PBX1* (pre-B-cell leukemia homeobox 1), *PDCD1LG2* (programmed cell death 1 ligand 2), *SLC2A1* [solute carrier family 2 (facilitated glucose transporter), member 1], *PUM2* (pumilio RNA-binding family member 2), *MET* (met proto-oncogene), *VCP* (Valosin Containing Protein) and *FUT8* [(fucosyltransferase 8 (alpha (1, 6) fucosyltransferase)] as potential gene targets of miR-198 predicted by all the four algorithms (**Supplementary Table S7**). Among these *FUT8*, *VCP*, *PUM2*, *PBX1* and *MET* are already explored targets of miR-198. A thorough literature search was performed for the remaining targets considering their impact in cancer, known role, implication in pathways etc. *TOPORS* is known to directly interact and regulate the expression of p53 (34). The role of TP53 in cancer progression is well established. Also, other pathways (MAPK, PI3K/AKT, HGF/MET, JAK/STAT and FGFR1) are already explored for miR-198 (27). However, none of the studies investigated the regulation of p53 after ectopic expression of miR-198. Thus, we decided to pursue *TOPORS* as a gene target for miR-198.

**Figure 3:**
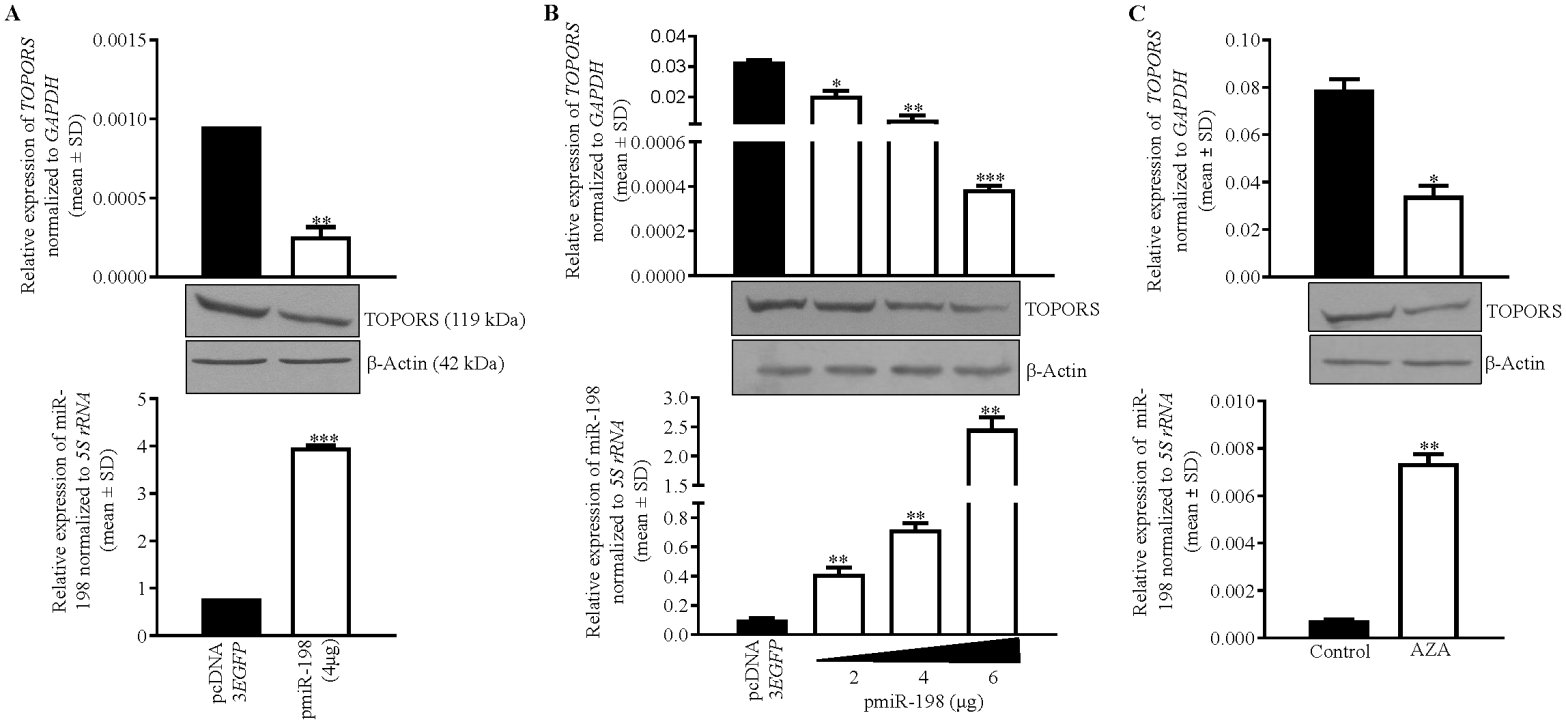
Identification of *TOPORS* as a gene target of miR-198. **(A)** Transfection of pmiR-198 in SCC131 cells shows decreased TOPORS transcript and protein levels compared with those transfected with the vector control. **(B)** A dose-dependent regulation of TOPORS by miR-198 in SCC131 cells. **(C)** The 5-Azacytidine treatment of SCC131 cells shows an increased level of miR-198 with concomitant decreased levels of TOPORS transcript and the protein. For qRT-PCR data,each bar is an average of 2 technical replicates.

To determine if miR-198 targets and regulates *TOPORS*, we transfected pmiR-198 and pcDNA3-EGFP separately in SCC131 cells and assessed the levels of TOPORS transcript and protein (**Figure 3A**). The results showed that miR-198 reduces the levels of TOPORS transcript and protein (**Figure 3A**), suggesting miR-198-mediated regulation of TOPORS. To determine if there is a dose-dependent regulation of TOPORS by miR-198, we transfected different quantities of the pmiR-198 construct in SCC131 cells. The results showed that miR-198 reduces the levels of TOPORS transcript and protein in a dose-dependent manner (**Figure 3B**). We next looked for the effect of 5-Azacytidine treatment on the expression of both TOPORS transcript and protein in SCC131 cells. The results showed a concomitant reduction in the levels of TOPORS transcript and protein with an increased level of miR-198 in 5-Azacytine treated cells compared with the control-treated cells (**Figure 3C**). These observations further strengthen the significance of miR-198-mediated regulation of TOPORS.

### 3.4 Confirmation of a direct interaction between miR-198 and the 3’UTR of *TOPORS*

**Figure 4:**
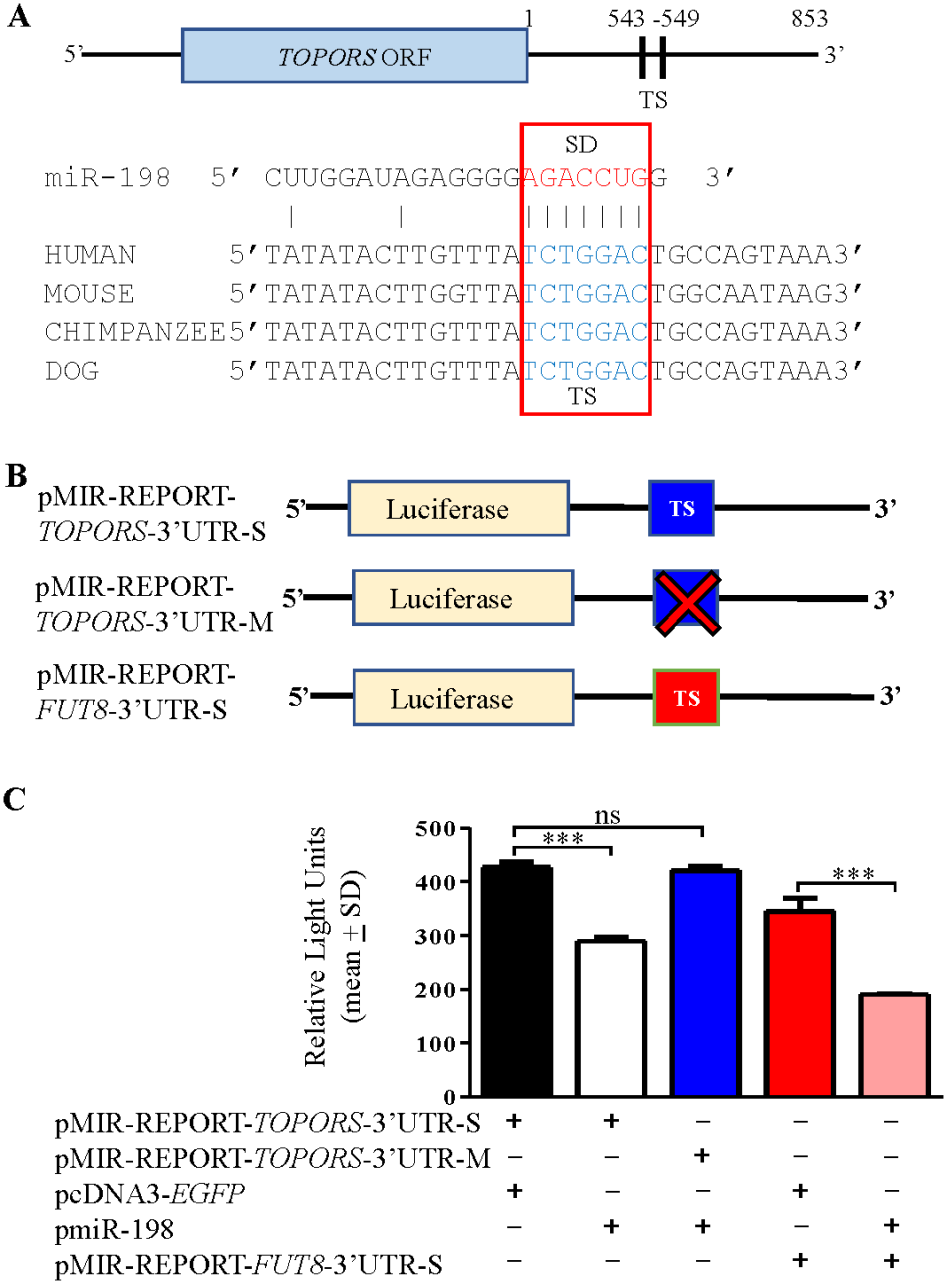
Confirmation of the binding of miR-198 to the 3’UTR of *TOPORS* by the dual-luciferase reporter assay in SCC131 cells. **(A)** Diagram showing conservation of the putative target site (TS) for the miR-198 seed sequence (SD) in the 3’UTR of *TOPORS* across species. The numbers denote nucleotide positions. **(B)** Schematic diagrams depicting various constructs used in the dual-luciferase reporter assay. The ‘X’ indicates abrogated TS region. **(C)** The results of dual-luciferase reporter assay. Note, a significantly reduced RLU in cells co-transfected with pMIR-REPORT-*TOPORS*-3’UTR-S and pmiR-198 compared to those transfected with pMIR-REPORT-*TOPORS*-3’UTR-S and pcDNA3-EGFP, confirming the binding of miR-198 to 3’UTR of *TOPORS*. Each bar is an average of 3 biological replicates.

The target prediction programs showed one putative target site (TS) in the 3’UTR of *TOPORS* from nucleotide positions 543-549 for binding of the miR-198 seed region (SD) (**Figure 4A**). The CLUSTALW alignment (https://www.genome.jp/tools-bin/clustalw) showed that this TS is conserved across species (**Figure 4A**). To validate the direct interaction between miR-198 and the 3’UTR of *TOPORS*, we employed the use of a gold standard assay, the dual-luciferase reporter (DLR) assay. The DLR assay was performed using various constructs illustrated in **Figure 4B**. Using the site-directed mutagenesis, we generated a negative control construct, pMIR-REPORT-*TOPORS*-3’UTR-M, by abrogating the TS for miR-198 in the 3’UTR of *TOPORS*. If miRNA binds to the 3’UTR of the target gene *TOPORS*, the luciferase activity significantly reduces compared with control transfected cells. Therefore, we co-transfected SCC131 cells with pMIR-REPORT-*TOPORS*-3’UTR-S containing the intact 3’UTR of *TOPORS* in a sense orientation and pmiR-198 or pMIR-REPORT-*TOPORS*-3’UTR-S and pcDNA3-*EGFP* (vector control) and quantified the luciferase reporter activity. We observed a significant reduction in the luciferase activity in cells co-transfected with pMIR-REPORT-*TOPORS*-3’UTR-S and pmiR-198 compared with those co-transfected with pMIR-REPORT-*TOPORS*-3’UTR-S and pcDNA3-*EGFP* (**Figure 4C**). As expected, cells co-transfected with pmiR-198 and pMIR-REPORT-*TOPORS*-3’UTR-M showed the luciferase activity comparable to those co-transfected with pMIR-REPORT-*TOPORS*-3’UTR-S and pcDNA3-*EGFP*. This is attributed to the lack of binding of miR-198 to the *TOPORS* 3’UTR, as the TS is abrogated in the pMIR-REPORT-*TOPORS*-3’UTR-M construct (**Figures 4B, C**). We also analysed the binding of miR-198 to its known gene target *FUT8* as a positive control. We observed that the cells co-transfected with pmiR-198 and pMIR-REPORT-*FUT8*-3’UTR-S showed a significantly reduced luciferase activity compared with those co-transfected with pMIR-REPORT-*FUT8*-3’UTR-S and pcDNA3-*EGFP*, confirming the binding of miR-198 to the 3’UTR of *FUT8*. The above observations confirm that miR-198 binds directly to the TS in the 3’UTR of *TOPORS* in a sequence-specific manner.

### 3.5 Expression analysis of *TOPORS* and miR-198 in OSCC patient samples

**Figure 5:**
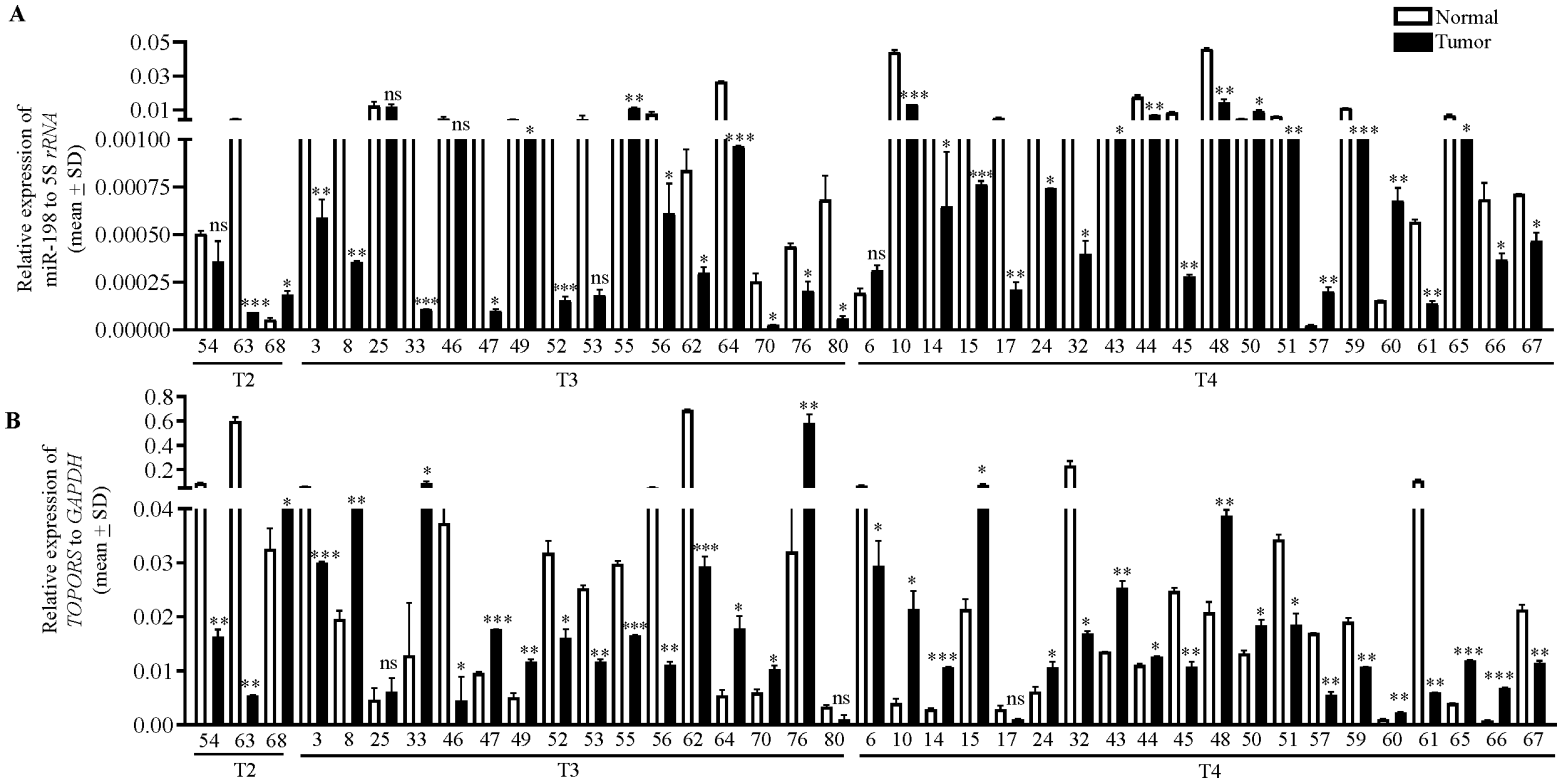
The qRT-PCR analysis of **(A)** miR-198 and **(B)** *TOPORS* in OSCC patient samples. The T2, T3 and T4 represent the different stages of tumors, and the numbers along X-axis denote different patient numbers. Each qRT-PCR data is an average of 2 technical replicates.

To understand the biological relevance of the interaction between miR-198 and TOPORS, we determined the levels of miR-198 and *TOPORS* transcripts in 39 matched normal oral tissue and OSCC patient samples, using qRT-PCR (**Figure 5**). We found miR-198 to be significantly downregulated in 29/39 (74.35%) OSCC samples compared with their matched normal oral tissue samples (viz., patient no. 63, 3, 8, 33, 47, 49, 52, 56, 62, 64, 70, 76, 80, 10, 14, 15, 17, 24, 32, 43, 44, 45, 48, 51, 59, 61, 65, 66 and 67) and significantly upregulated in tumor samples compared with their matched normal oral tissue samples in 5/39 (12.8%) (viz., patient no. 68, 55, 50, 57 and 60) (**Figure 5A**). Moreover, no significant change in its levels was observed in 5/39 (12.8%) OSCC samples compared with their normal tissue counterparts (viz., patient no. 54, 25, 46, 53 and 6). Further, we found *TOPORS* to be significantly upregulated in 19/39 (48.7%) OSCC samples compared with their matched normal oral tissue samples (viz., patient no. 68, 8, 33, 47, 49, 64, 70, 76, 10, 14, 15, 24, 43, 44, 48, 50, 60, 65 and 66) (**Figure 5B**) and significantly downregulated in 17/39 (43.58%) OSCC samples compared with their matched normal oral tissues (viz., patient no 54, 63, 3, 46, 52, 53, 55, 56, 62, 6, 32, 45, 51, 57, 59, 61 and 67) (**Figure 5**). Moreover, no significant change was reported in its levels in 3/39 (7.6%) (viz., patient no. 25, 80 and 17) OSCC samples compared with their matched normal tissue samples (**Figure 5B**). In patient numbers 8, 33, 47, 49, 64, 70, 76, 10, 14, 15, 24, 43, 44, 48, 65, and 66 the miR-198 expression is significantly downregulated compared with their matched normal oral tissue samples and concomitantly the *TOPORS* expression is significantly upregulated compared with their matched normal oral tissue samples. In patient numbers 55 and 57 miR-198 is significantly upregulated compared with their matched normal oral tissue samples and concomitantly *TOPORS* is significantly downregulated compared with their matched normal oral tissue samples. Overall, an inverse relation of such kind was observed between the levels of miR-198 and *TOPORS* in 18/39 (46.15%) (viz., patient no. 8, 33, 47, 49, 55, 64, 70, 76, 10, 14, 15, 24, 43, 44, 48, 57, 65 and 66) OSCC patient samples (**Figure 5**), suggesting the biological relevance of their interaction.

### 3.6 TOPORS overexpression enhances OSCC cell proliferation

MiRNAs are known to negatively regulate their target genes by binding to their 3’UTRs. As we have observed miR-198 to be a tumor suppressor miRNA, which targets *TOPORS*, it is interesting to study the role of TOPORS in OSCC cells. To this end, we transfected SCC131 and SCC084 cells with vector control and p*TOPORS* separately and checked for cell proliferation by trypan blue dye exclusion assay. We observed a significant increase in proliferation of cells transfected with p*TOPORS* compared with those transfected with the vector control, suggesting that TOPORS exerts a positive regulation on proliferation of OSCC cells from both cell lines (**Supplementary Figure S2**).

### 3.7 Expression of *TOPORS* depends on the presence or absence of its 3’UTR

Since miR-198 binds directly to the 3’UTR of *TOPORS*, we wanted to investigate if the function of TOPORS depends on the presence of its 3’UTR. To this end, we utilized the following different *TOPORS* overexpression constructs: p*TOPORS* harbouring a complete *TOPORS* ORF, p*TOPORS*-3’UTR-S with the 3’UTR of *TOPORS* cloned downstream to the *TOPORS* ORF in the p*TOPORS* construct in a sense orientation and p*TOPORS*-3’UTR-M with the abrogated TS in the 3’UTR of *TOPORS* cloned downstream to the *TOPORS* ORF in the p*TOPORS* construct. We then transfected the vector control [pcDNA3.1(+)] only or co-transfected vector control and pmiR-198 or pmiR-198 with pTOPORS, pTOPORS-3’UTR-S or pTOPORS-3’UTR-M in SCC084 and SCC131 cells and performed Western blot analysis to assess TOPORS levels (**Supplementary Figure S3**). The results showed that the TOPORS levels is reduced in cells co-transfected with the vector and pmiR-198 compared with those transfected with the vector control only, because of miR-198 targeting the endogenous TOPORS (**Supplementary Figure S3**). Also, an increased level of TOPORS was observed in cells co-transfected with pmiR-198 and p*TOPORS* as compared to those co-transfected with the vector and pmiR-198 (**Supplementary Figure S3**). Further, we observed a reduced level of TOPORS in cells co-transfected with p*TOPORS*-3’UTR-S and pmiR-198 as compared with those co-transfected with p*TOPORS* and pmiR-198, due to the presence of a functional TS in the 3’UTR of p*TOPORS*-3’UTR-S (**Supplementary Figure S3**). Further, an increased level of TOPORS was observed in cells co-transfected with p*TOPORS*-3’UTR-M and pmiR-198 as compared with those co-transfected with p*TOPORS*-3’UTR-S and pmiR-198, because of the absence of TS in the 3’UTR of p*TOPORS*-3’UTR-M (**Supplementary Figure S3**). These observations suggest that TOPORS expression depends on the presence or absence of its 3’UTR and is, in part, regulated by miR-198.

### 3.8 miR-198 regulates proliferation, apoptosis and anchorage-independent growth of OSCC cells, in part, via targeting the 3’UTR of *TOPORS*

**Figure 6:**
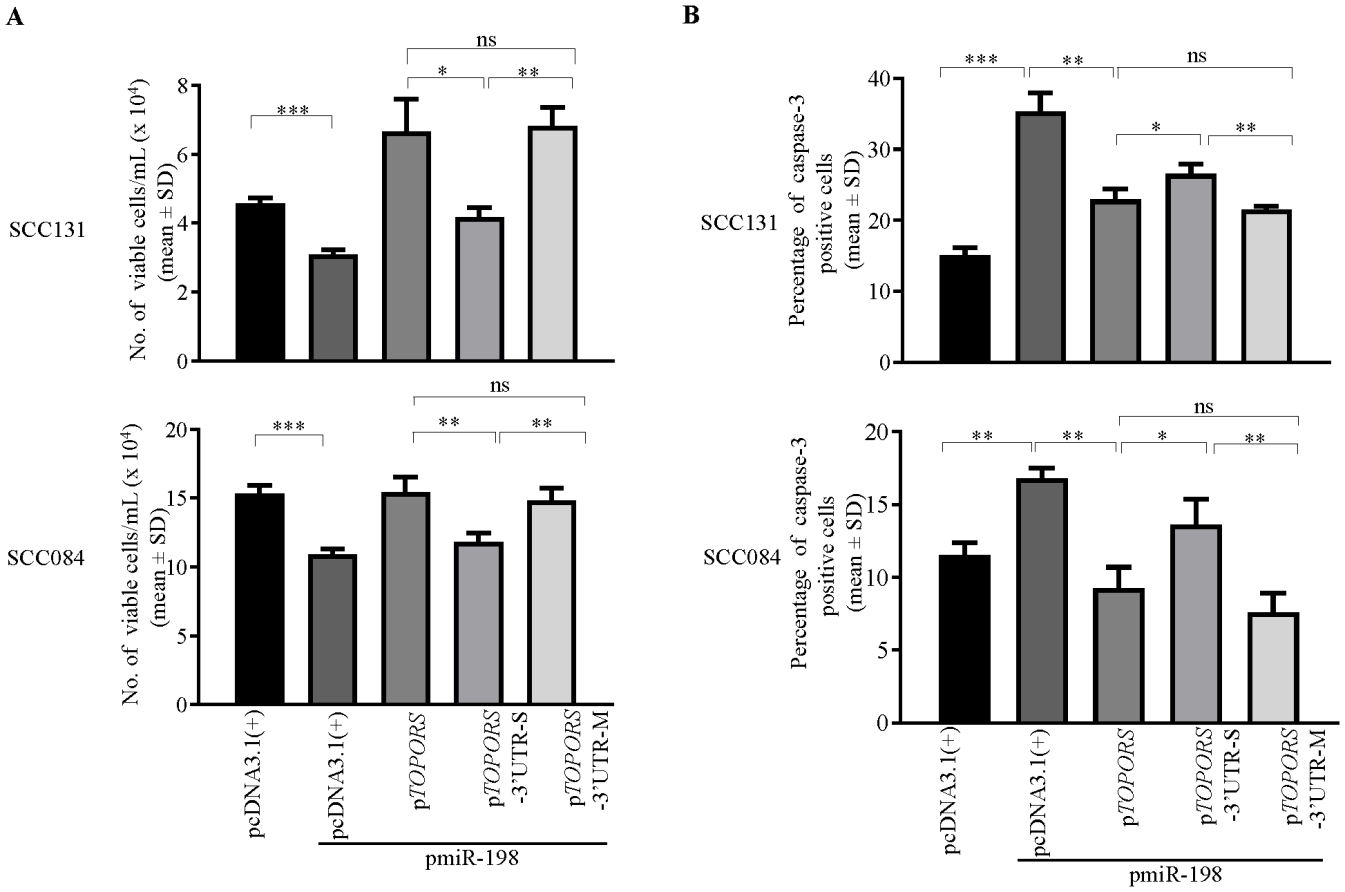
Regulation of cell proliferation and apoptosis by miR-198, in part, via targeting the 3’UTR of *TOPORS*. **(A)** The analysis of cell proliferation by the trypan blue dye exclusion assay in SCC131 and SCC084 cells co-transfected with pmiR-198 and different TOPORS overexpression constructs or vector control. Note, a significantly decreased rate of proliferation of cells co-transfected with p*TOPORS*-3’UTR-S and pmiR-198 compared to those co-transfected with p*TOPORS* and pmiR-198 or p*TOPORS*-3’UTR-M and pmiR-198. **(B)** The quantitative analysis of apoptosis rate as assessed by the caspase-3 assay in SCC131 and SCC084 cells co-transfected with pmiR-198 and different TOPORS overexpression constructs or vector control. Note, a significant increase in the rate of apoptosis on overexpression of pmiR-198 compared with the vector control in both SCC131 and SCC084 cells. Also, there is a significant increase in the rate of apoptosis of cells co-transfected with p*TOPORS*-3’UTR-S and pmiR-198 in comparison to those co-transfected with p*TOPORS* and pmiR-198 or p*TOPORS*-3’UTR-M and pmiR-198. Each bar is an average of 3 biological replicates.

We then used the above constructs to assess the effect of miR-198 mediated regulation of TOPORS on a few hallmarks of cancer such as cell proliferation, apoptosis, and anchorage-independent growth. To this end, we co-transfected p*TOPORS*, p*TOPORS*-3’UTR-S and p*TOPORS*-3’UTR-M constructs separately with pmiR-198 in SCC131 and SCC084 cells and assessed cell proliferation using the trypan blue dye exclusion assay. As expected, we observed a decreased rate of cell proliferation in cells co-transfected with pmiR-198 and vector compared with those transfected with the vector only (**Figure 6A**), due to targeting of endogenous TOPORS by miR-198. Further, we observed a decreased rate of cell proliferation in cells co-transfected with pmiR-198 and pTOPORS-3’UTR-S compared with those co-transfected with pmiR-198 and pTOPORS, due to the presence of a functional TS in the 3’UTR of pTOPORS-3’UTR-S (**Figure 6A**). As expected, we observed no difference in the rate of cell proliferation in cells co-transfected with pmiR-198 and p*TOPORS* compared with those co-transfected with pmiR-198 and p*TOPORS*-3’UTR-M, due to the absence of a functional TS in 3’UTR of pTOPORS-*3’UTR*-M (**Figure 6A**). A similar observation was made in cells from both cell lines SCC131 and SCC084 (**Figure 6A**). These observations highlighted the negative regulation of cell proliferation by miR-198, in part, via targeting the 3’UTR of *TOPORS*.

To determine if miR-198 regulates apoptosis by targeting the 3’UTR of *TOPORS*, we again used the above constructs and performed caspase-3 assay and analysed the percentage of caspase-3 positive cells in SCC131 and SCC084 cells. We observed a significant increase in the rate of apoptosis in cells co-transfected with pmiR-198 and vector control compared with those transfected with vector control only (**Figure 6B**). Also, the percentage of apoptotic cells was significantly higher in cells co-transfected with p*TOPORS*-3’UTR-S and pmiR-198 compared with those co-transfected with p*TOPORS*-3’UTR-M or p*TOPORS* and pmiR-198, due to binding of the miR-198 to the TS in p*TOPORS*-3’UTR-S (**Figure 6B**). The percentage of caspase-3 positive cells was significantly higher in cells transfected pmiR-198 and pcDNA3.1(+) compared with those co-transfected with p*TOPORS* and pmiR-198, due to ectopic expression of oncogenic *TOPORS*-ORF (**Figure 6B**). These observations suggested that miR-198 positively regulates cellular apoptosis in SCC131 and SCC084 cells, in part, by directly targeting the 3’UTR of *TOPORS*.

**Figure 7:**
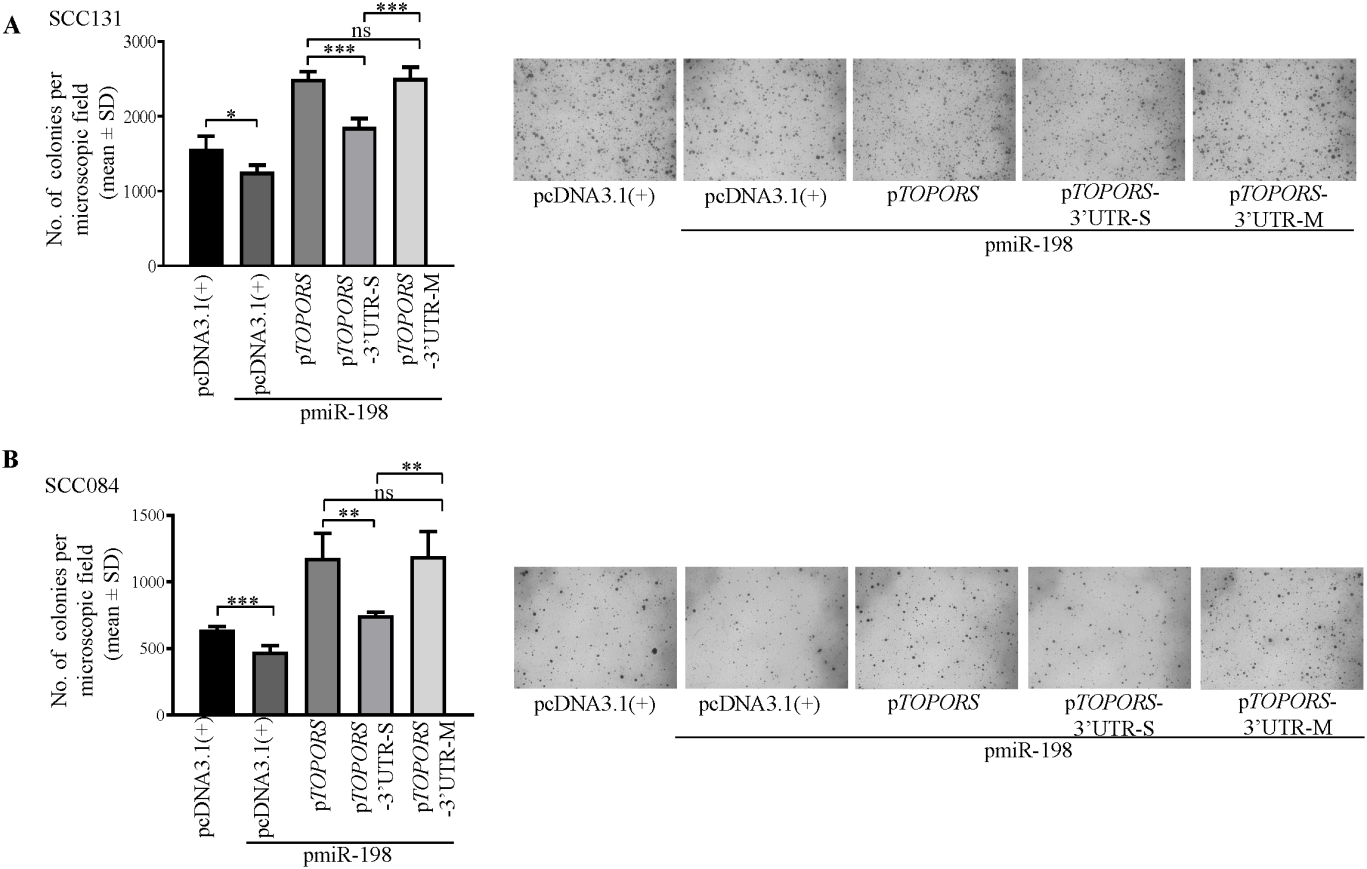
Regulation of anchorage-independent growth of OSCC cells by miR-198, in part, via targeting the 3’UTR of *TOPORS*. Quantitative assessment of the anchorage independent growth capabilities and representative microphotographs of the colonies for **(A)** SCC131 and **(B)** SCC084 cells co-transfected with pmiR-198 and different TOPORS overexpression constructs or vector control by soft agar colony forming assay. Each bar is an average of 4 biological replicates.

To investigate the effect of miR-198-mediated regulation of *TOPORS* on anchorage-independent growth of OSCC cells, we co-transfected p*TOPORS*, p*TOPORS*-3’UTR-S and p*TOPORS*-3’UTR-M separately with pmiR-198 in SCC084 and SCC131 cells by soft agar colony forming assay (**Figure 7**). As expected, we observed a reduced number of colonies in cells co-transfected with pmiR-198 and the vector compared with those transfected with the vector only (**Figure 7**). Further, a reduced number of colonies was observed in cells co-transfected with pmiR-198 and p*TOPORS*-3’UTR-S compared with those co-transfected with p*TOPORS* and pmiR-198 (**Figure 7**), due to binding of miR-198 to the TS in the p*TOPORS*-3’UTR-S. Further, no difference in the number of colonies was observed in cells co-transfected with pmiR-198 and p*TOPORS* compared with those co-transfected with pmiR-198 and p*TOPORS*-3’UTR-M, due to the absence of a functional TS in p*TOPORS*-3’UTR-M (**Figure 7**). These results clearly suggested a negative regulation of miR-198 on the anchorage-independent growth, in part, by targeting the 3’UTR of *TOPORS*.

### 3.9 Restoration of miR-198 levels by a synthetic mimic suppresses tumor growth *in vivo*

**Figure 8:**
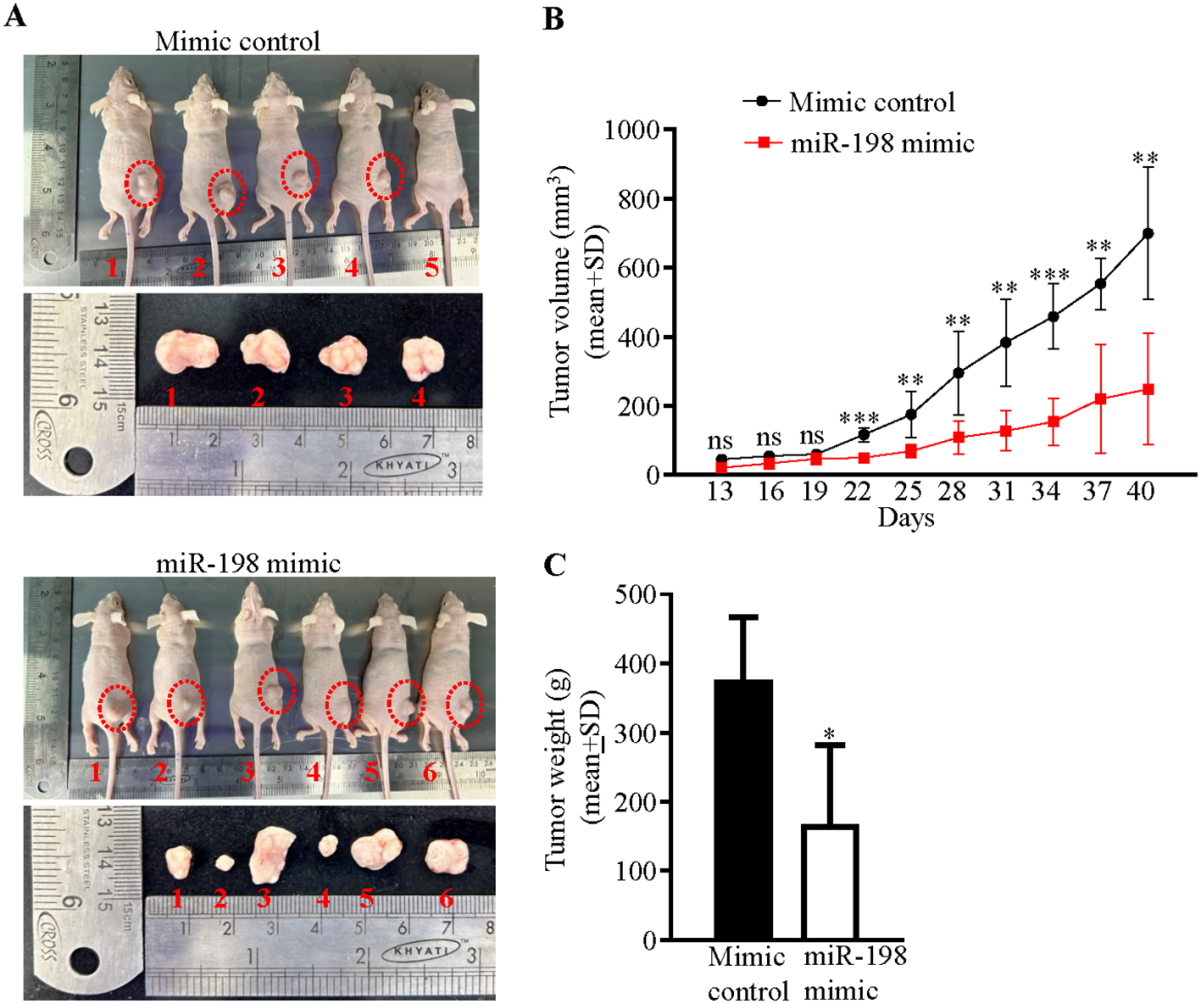
Restoration of miR-198 level suppresses tumorigenesis *in vivo*. **(A)** Photographs of nude mice showing tumor growth on day 40 of injection. The numbers denote mice numbers. The corresponding excised xenograft on day 40 is shown below each mouse. Each group had 6 mice. One mouse (#6) in the mimic control group died early. Dotted red circles mark the xenografts in mice. Note, no tumor development in mice # 5 in the mimic control group. **(B)** Effect of miR-198 mimic on the volume of xenografts during a time course of 40 days. **(C)** The weight of xenografts on day 40.

Since we had observed an anti-proliferative role of miR-198, we hypothesized that the restoration of the expression level of the miR-198 by a synthetic miR-198 mimic could reduce the level of oncogenic TOPORS in OSCC cells, which might have an anti-tumor effect *in vivo*. To this end, we used *in vivo* pre-treated OSCC xenograft nude mouse model. We first optimized the dosage of a synthetic miR-198 mimic in SCC131 cells and found 900 nM of the mimic to be an optimum dose to reduce TOPORS at both the transcript and protein levels (**Supplementary Figure S4**). We then injected equal numbers of pre-transfected SCC131 cells with 900 nM of miR-198 mimic (M900) or 900 nM of mimic control (MC900) separately into the right flanks of female nude mice (6 weeks of age). The mice were monitored for OSCC xenograft (tumor) growth for 40 days. As expected, there was a reduction in both tumor volume and weight in nude mice injected with miR-198 mimic treated cells compared to that of the mock control (**Figure 8**), suggesting that miR-198 functions as tumor suppressor in OSCC.

### 3.10 Effect of miR-198-mediated regulation of TOPORS on the p53/p21 signaling in OSCC cells

**Figure 9:**
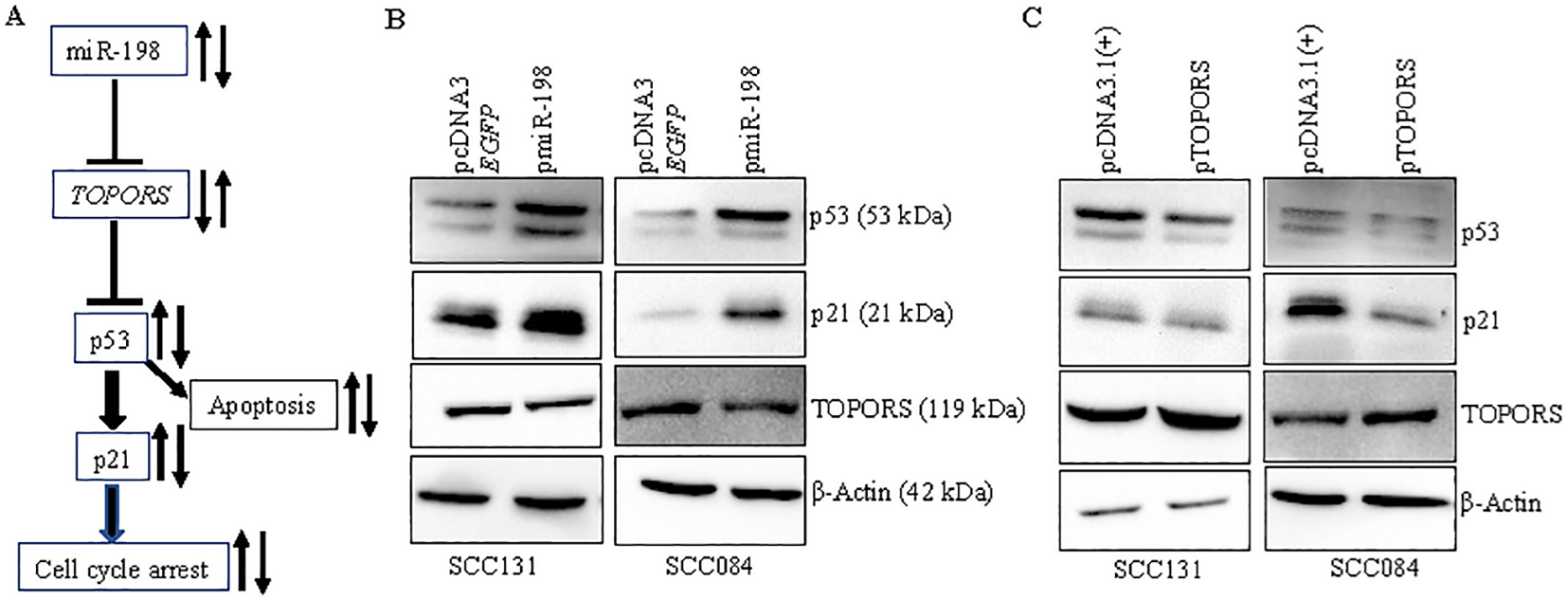
miR-198 upregulates p53/p21 signaling, in part, by regulating TOPORS. **(A)** A diagrammatic representation of the miR-198/TOPORS/p53/p21 axis. **(B)** Western blot analysis following overexpression of miR-198 in SCC131 and SCC084 cells. **(C)** Western blot analysis following overexpression of TOPORS in SCC131 and SCC084 cells. β-actin was used as a loading control.

TOPORS, an E3 ubiquitin and SUMO ligase, is known to affect the expression of several proteins, including the guardian of the genome, p53 (34–38). The p53 pathway is well known for induction of apoptosis and cell cycle arrest (39). The effect of miR-198 on the p53 signaling has not been explored. We, therefore, wanted to understand if miR-198-mediated regulation of TOPORS has any impact on the p53 pathway in OSCC (**Figure 9A**). To this end, we determined the levels of p53 and its downstream target p21 (WAF1/CIP1/CDKN1A) in OSCC cells transfected with pmiR-198 or p*TOPORS*, using the Western blot analysis (**Figures 9B, C**). The results showed that the cells transfected with pmiR-198 had increased levels of p53 and p21 compared with those transfected with the vector control in both SCC131 and SCC084 cells (**Figure 9B**). As expected, overexpression of miR-198 led to a reduced level of TOPORS in both SCC131 and SCC084 cells (**Figure 9B**). In contrast, cells transfected with p*TOPORS* showed reduced levels of p53 and p21 compared with those transfected with the vector control in both SCC131 and SCC084 cells (**Figure 9C**). These results highlighted the significant roles miR-198 and TOPORS in the p53 pathway in OSCC. These observations also indicated that miR-198 enhances signaling of p53/p21, in part, by regulating TOPORS.

### 3.11 Effect of miR-198-mediated regulation of *TOPORS* and p53/p21 signalling in OSCC patient samples

**Figure 10:**
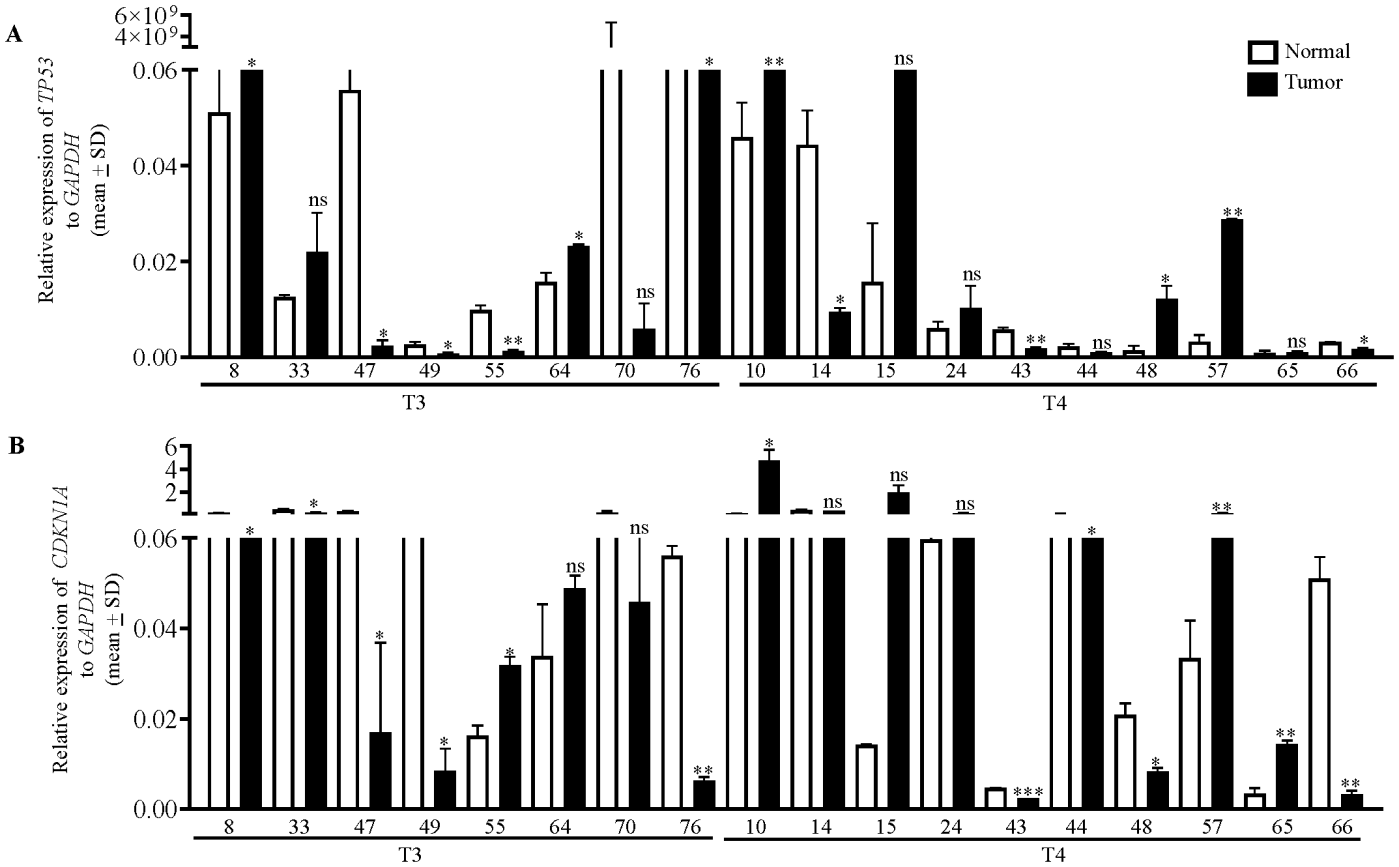
The qRT-PCR analysis of *TP53* and *CDKN1A* in OSCC patient samples. The transcript levels of **(A)** *TP53* and **(B)** *CDKN1A* in 18 matched normal oral tissue and OSCC tissue samples from patients who showed an inverse correlation in the levels of miR-198 and *TOPORS*. T3 and T4 represent the different stages of tumors, and the numbers along X-axis denote different patient numbers. Each qRT-PCR data is an average of 2 technical replicates.

Since the p53/p21 pathway is observed to be directly correlated with miR-198-mediated regulation of *TOPORS* (**Figure 9**), we wanted to explore the levels of *TP53* transcript in the OSCC patient samples which showed an inverse relation of miR-198 and *TOPORS* levels (viz., patient no. 8, 33, 47, 49, 55, 64, 70, 76, 10, 14, 15, 24, 43, 44, 48, 57, 65 and 66) by qRT-PCR using *TP53* specific primers (**Supplementary Table S3**). The results showed that *TP53* was significantly downregulated in 7/18 OSCC samples (viz., patient no. 47, 49, 55, 76, 14, 43 and 66) and upregulated in 5/18 OSCC samples (viz., patient no. 8, 64, 10, 48 and 57) compared with their matched normal tissue samples (**Figure 10**). Since *TP53* is the most mutated gene in cancer, we checked the *CDKN1A* transcript level using *CDK1NA* (*P21*) specific primer (**Supplementary Table S3**) as mutations in *CDKN1A* are less frequent and *CDKN1A* is a direct target and a downstream effector for p53. The results showed a significant downregulation of the *CDKN1A* level in 9/18 OSCC samples (viz., patient no. 8, 33, 47, 49, 76, 43, 44, 48 and 66) and its upregulation in 4/18 OSCC samples (viz., patient no. 55, 10, 57 and 65) compared to matched normal oral tissue samples (**Figure 10**). No significant change between their levels was observed in 6/18 OSCC samples for *TP53* (viz., patient no. 33, 70, 15, 24, 44 and 65) and 5/18 OSCC samples for *CDKN1A* (viz., patient no. 64, 70, 14, 15 and 24) compared with matched normal oral samples (**Figure 10**). We then analyzed the expression pattern of *TP53* and *CDKN1A* with respect to miR-198 expression in these inversely related patient samples. Interestingly, we observed miR-198 levels to be positively correlated with *CDKN1A* levels in 11/18 OSCC samples compared with their matched normal oral tissue samples (viz., patient no. 8, 33, 47, 49, 55, 76, 43, 44, 48, 57 and 66) (**Figure 10**). For example, if miR-198 expression is observed to be downregulated in the tumor sample from patient no. 47, the expression of *CDKN1A* (downstream effector of p53) and *TP53* was also downregulated and the expression of *TOPORS* was upregulated in this tumor sample compared with its matched normal tissue sample (**Figure 10**). The Pearson correlation analysis indicate an inverse correlation between miR-198 and *TOPORS* (r = -0.08; p value=0.614) whereas the analysis of miR-198 and *CDKN1A* (r = 0.422; p value=0.080) indicate a positive correlation (**Supplementary Figure S5**) but the correlation was not significant. The small sample size, tumor heterogeneity and the presence of other underlying alternate mechanisms are possible reasons. Nonetheless, these results highlighted the biological relevance of this interaction between miR-198 and *TOPORS* and its role in the p53/p21 pathway.

## 4 Discussion

Epigenetic silencing is one of the major mechanisms that regulate tumor suppressor miRNAs and induce tumor progression (40). To our knowledge, very few miRNAs (e.g., miR-137, miR-127, miR-375, miR-34b, miR-193a, miR-200/miR-205, miR-6741-3p, and miR-617) are known to be regulated by promoter hypermethylation in OSCC (13, 14, 26, 41). Here we report for the first time that miR-198 is an epigenetically silenced tumor suppressor miRNA in OSCC. We identified an independent promoter for miR-198 and using BSP, we established that miR-198 expression increases following 5-Azacytidine (hypomethylating drug) treatment due to demethylation at the *MIR198* promoter in OSCC (**Figures 1A**, **2B**). Contrary to our observations, Sundaram et al. (42) identified a bi-directional switch *FSTL1*/miR-198 as a mechanism for transcriptional regulation of miR-198. The regulation of exonic miRNAs such as miR-198 is not well understood and elucidated as they are relatively low in number compared with intronic miRNAs (43). Some miRNAs are also known to be under dual regulation of their host gene promoters and their independent promoters (44–46). To make matters even more complex, miR-198 is known to be regulated by several non-coding RNAs (27), highlighting the varied ways that miRNAs are regulated and how we still require additional studies to understand this complex interaction.

miRNAs regulate gene expression by modulating the expression of their target genes. We showed for the first time that the overexpression of miR-198 reduces the expression of its target *TOPORS* at both transcript and protein levels in OSCC cells by directly binding to its 3’UTR in a sequence-specific manner (**Figure 4**). Though 45 gene targets for miR-198 are reported across various cancers, the role of only miR-198 mediated regulation of *CDK4* is reported in OSCC (4, 27). It is interesting to note that, till date no other miRNA is known to target *TOPORS*. Also, we show here for the first time the oncogenic role of TOPORS in OSCC.

miR-198, an exonic miRNA located in the last non-coding exon 11 of the *FSTL1* (Follistatin Like 1) gene on chromosome 3q13.33, acts as a tumor suppressor in several cancers (27). Wang et al. (47) observed that downregulation of miR-198 is significantly associated with tumor size, lymph node metastasis, and tumor node metastasis (TNM) stage in patients with non-small cell lung cancer (NSCLC). TOPORS is a multifunctional protein belonging to the RING family of proteins and functions as both E3 ubiquitin ligase and E3 SUMO ligase. It is involved in several cellular processes such as maintenance of retinal homeostasis, cell division, maintenance of genomic stability, chromatin organization, double strand break repair and base excision repair (34–38). In accordance with other studies showing tumor suppressive function of miR-198, we show that TOPORS is upregulated and miR-198 is downregulated in a majority of OSCC tumors compared to their normal counterparts (**Figure 5**). However, we did not observe any correlation in 21/39 OSCC samples, suggesting the involvement of alternate mechanisms such as tumor heterogeneity and heterogenous genetic composition of each patient (14, 48). An inverse correlation between the expression levels of miR-198 and TOPORS in a majority of OSCC samples suggests the biological significance of their interaction.

Previous studies have explored the involvement of miR-198 in MAPK and PI3K/AKT, HGF/MET, JAK/STAT and FGFR1 pathways (27). Further, TOPORS is known to directly interact and regulate the expression of p53 (38). However, none of the studies have investigated a link between miR-198 and p53. Thus, we decided to focus on this pathway and investigated the levels of p53 and its downstream effector target p21 (CDKN1A/CIP1/WAF1) following miR-198 and TOPORS overexpression in OSCC cells. The miR-198 overexpression should reduce TOPORS level. A reduction in TOPORS expression is expected to overcome p53 inhibition, subsequently increasing p53 and p21 levels. As expected, we observed reduced TOPORS levels and enhanced p53 and p21 levels in miR-198 overexpressed cells from both OSCC cell lines and vice-versa for TOPORS overexpressed cells (**Figure 9**). In the OSCC patient samples which showed an inverse correlation between miR-198 and TOPORS expression (18 patients) (**Figure 5**), we also observed the miR-198 levels to be positively correlated with CDKN1A (p21) levels in 11/18 tumor samples (viz., patient no. 8, 33, 43, 44, 47, 48, 49, 55, 57, 66 and 76) compared with their matched normal oral samples (**Figures 5**, **10**), highlighting the biological relevance of miR-198-mediated TOPORS regulation on the p53-p21 signalling pathway.

miR-198 deregulation has been linked to modulation of various essential cellular properties, including proliferation, apoptosis, colony-forming ability, and invasion in a wide range of cancers such as prostate, gastric, lung, hepatocellular, and breast cancers (27). Interestingly, as it is known that a single miRNA can target many genes and a gene can also be targeted by many miRNAs (49), we therefore wanted to confirm that miR-198-mediated regulation of TOPORS is due the interaction between the 3′UTR of *TOPORS* and miR-198. To achieve this goal, we have transfected p*TOPORS*, p*TOPORS*-3′UTR-S, and p*TOPORS*-3′UTR-M constructs separately with pmiR-198 in OSCC cells. As expected, we observed a significant decrease in cell proliferation (**Figure 6A**) and anchorage-independent growth (**Figure 7**), and a significant increase in apoptosis (**Figure 6B**) in OSCC cells co-transfected with p*TOPORS*-3′UTR-S and pmiR-198 as compared to those co-transfected with p*TOPORS* or p*TOPORS*-3′UTR-M and pmiR-198, confirming that miR-198 regulates various hallmarks of cancers, in part, by directly targeting the 3′UTR of *TOPORS*.

**Figure 11:**
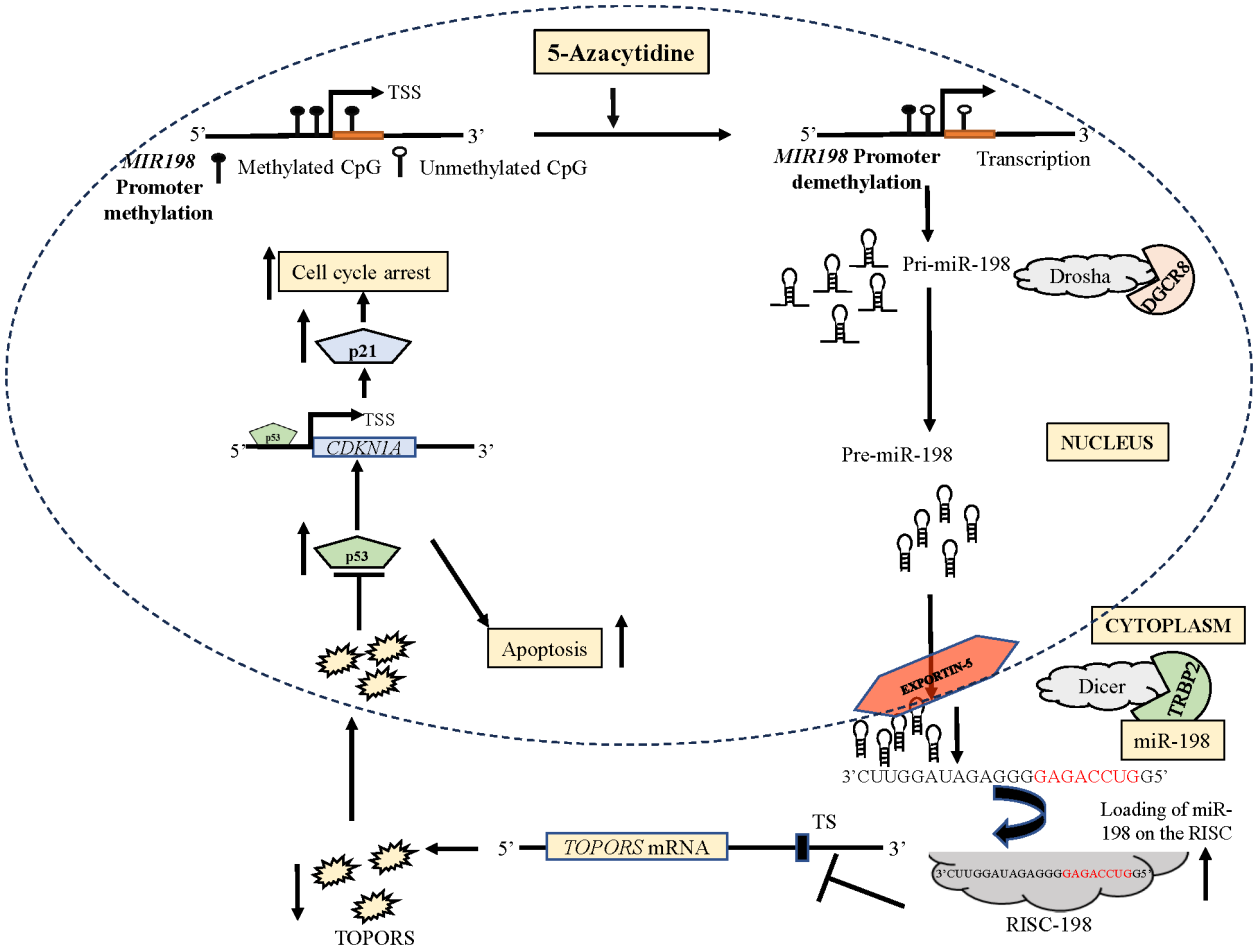
A diagrammatical representation of the effect of 5-Azacytidine induced upregulation of miR-198 in SCC131 cells.The independent promoter for miR-198 is represented by three CpG sites. The treatment of 5-Azacytidine of SCC131 cells leads to demethylation of the *MIR198* promoter. This causes an increase in the expression of primary miR-198 (Pri-miR-198) due to an increased transcription of miR-198. The Pri-miR-198 is processed by Drosha/DGCR8 complex to form the preliminary miR-198 (Pre-miR-198). The Pre-miR-198 is then exported out of the nucleus to the cytoplasm by Exportin-5. In the cytoplasm, it is processed for maturation via the Dicer/TRBP complex. The mature miR-198 is loaded on the RISC. This miR-198-RISC then binds to the TS on the 3’UTR of *TOPORS* mRNA, causing reduced expression of TOPORS. Reduced expression of TOPORS in turn increases p53 expression, causing apoptosis. The p53 also enhances the expression of p21 and subsequently increases cell cycle arrest. RISC, RNA-induced silencing complex; TS, target site; TSS, transcription start site.

Several studies reported reduced tumor size and volume in nude mice injected with miR-198 synthetic mimics compared with control group (50, 51). Kang et al. (4) injected athymic mice with OSCC cells (Cal-27-tongue cancer cell line) transfected with a synthetic miR-198 mimic and reported a significant reduction in tumor size and volume compared with mice injected with a mimic control. In our study, the mice injected with a miR-198 synthetic mimic treated OSCC cells showed reduced tumor weight and volume compared to mock treated cells, which reveals the tumor suppressor function of miR-198 (**Figure 8**). Taken together, these results suggested that miR-198 exerts its tumor suppressor functions in OSCC at least, in part, by regulating TOPORS (**Figure 11**), and therefore synthetic miR-198 mimics could be used as a therapeutic target in oral cancer However, the limitations of such study include the lack of a more diverse patient cohort for miR-198 and *TOPORS* expression analysis, the need for protein interaction studies between TOPORS and p53 in patient samples where a correlation was observed, the exploration of the combinatorial effect of miR-198 and its other known targets with TOPORS in OSCC, and the investigation of the significance of individual CpG sites in the *MIR198* promoter that contribute to epigenetic silencing. Further research is required to deepen our understanding of miR-198 regulation in these contexts.

Despite their small size, miRNAs play a pivotal role in regulating numerous cellular processes. Tumor suppressor and oncogenic miRNAs, along with their various targets influencing multiple pathways, present new opportunities for therapeutic interventions (52). Therapies may focus on restoring tumor suppressor miRNAs using miRNA mimics or inhibiting oncogenic miRNAs with miRNA inhibitors. Currently, several pharmaceutical companies are competing to develop effective delivery systems, with miRNAs such as miR-155, miR-10b, and miR-16 undergoing clinical trials for T-cell lymphoma, glioma, and lung cancer, respectively (52). These promising advancements could pave the way for personalized cancer treatments in future.

## 5 Conclusions

Our study has identified that miR-198 is an epigenetically silenced tumor suppressor, which directly targets and represses TOPORS and thereby activates the p53/p21 signaling in OSCC cells. Further, our observations have shown that miR-198 suppresses cell proliferation, anchorage-independent growth and enhances apoptosis of OSCC cells, in part, by targeting the 3’UTR of *TOPORS*. Moreover, our observations in nude mice highlight the therapeutic potential of miR-198 and TOPORS in OSCC. An in-depth understanding of the mechanisms by which miR-198 affects cancer progression could pave the way for miR-198 to serve as a therapeutic target for not just oral cancer but other cancers as well.

## Data Availability

The original contributions presented in the study are included in the article/**Supplementary Material**. Further inquiries can be directed to the corresponding author/s.
