# Supplementary material for: miR-198 targets TOPORS: implications for oral squamous cell carcinoma pathogenesis
Source: Front Oncol. 2024 Dec 4;14:1485802. doi: 10.3389/fonc.2024.1485802 (PMC11652479; doi:10.3389/fonc.2024.1485802)
Supplement: Supplementary file 1 [file DataSheet1.pdf]

Supplementary Materials

miR-198 targets *TOPORS*: implications for oral squamous cell carcinoma pathogenesis

Pankhuri Kaushik[1*], Radha Mishra[1], Champaka Gopal[2] and Arun Kumar[1*]

[1]Department of Developmental Biology and Genetics, Indian Institute of Science, Bangalore 560012, India

[2]Department of Pathology, Kidwai Memorial Institute of Oncology, Bangalore 560022, India

*Corresponding authors:

Email: pankhuri000@gmail.com

Email ID: arunk@iisc.ac.in

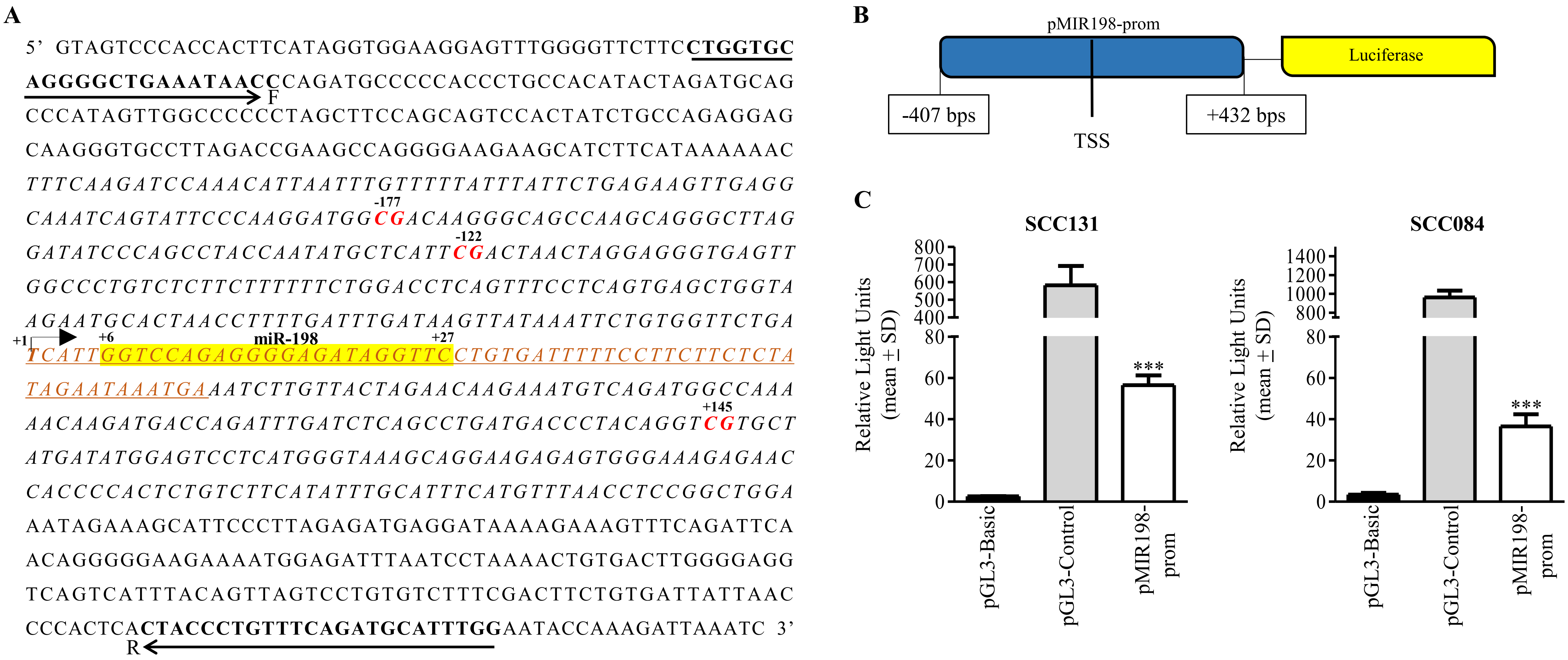

Supplementary Figure S1. Identification of the *MIR198* promoter. **(A)** The promoter region of *MIR198* predicted by the DBTSS database along with additional upstream and downstream sequences retrieved from the Switchgear and MatInspector databases. The transcription start site (TSS) is numbered as +1, and the rest of the sequence is numbered relative to it. The pre-miRNA sequence of miR-198 is represented as underlined orange colour. The mature miR-198 sequence is highlighted in yellow. The predicted CpG sites are coloured red. The sequence predicted by DBTSS is in italics. **(B)** A schematic diagram of the putative *MIR198* promoter construct pMIR-198-prom. **(C)** The dual-luciferase reporter assay in SCC131 and SCC084 cells showed a significant promoter activity for the *MIR198* promoter construct (pMIR198-prom). The pGL3-Control harbours the SV40 promoter and was used as a positive control, and pGL3-Basic has no promoter and was used as a negative control. Each bar is an average of 4 biological replicates.

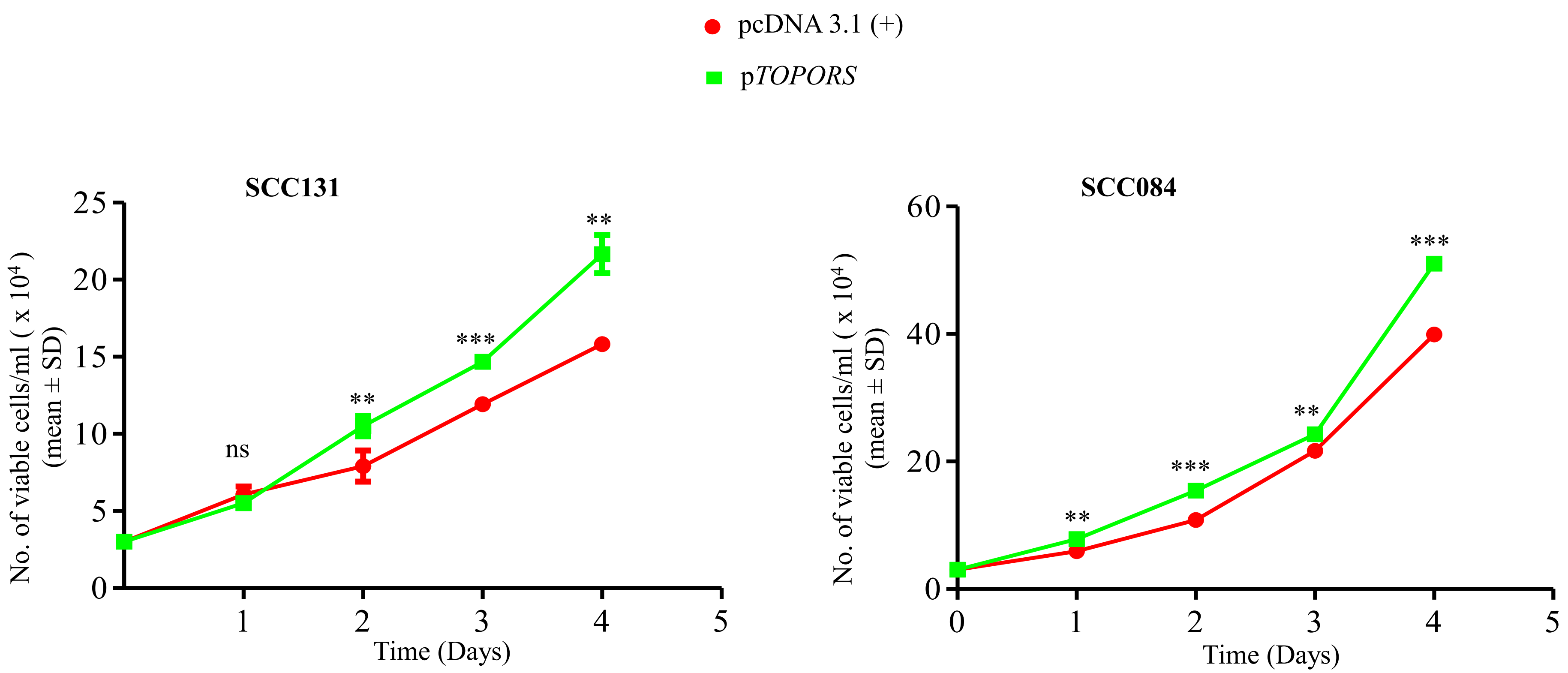

Supplementary Figure S2. Effect of *TOPORS* overexpression on proliferation of SCC131 and SCC084 cells. Note, a significant increase in proliferation of SCC131 and SCC084 cells transfected with p*TOPORS* compared to those transfected with the vector control. Each data point is an average of 3 biological replicates.

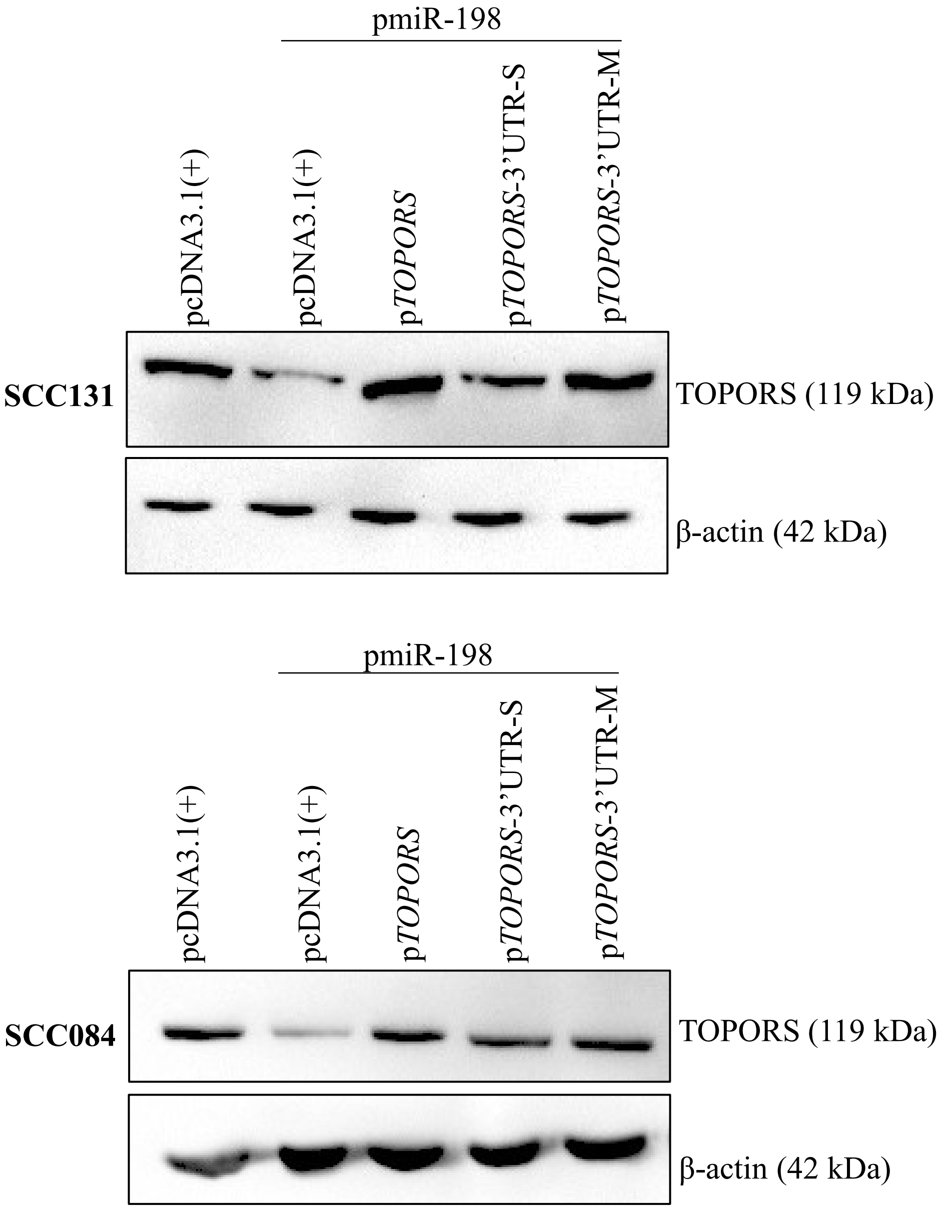

Supplementary Figure S3. TOPORS expression depends on the presence or absence of its 3'UTR. The Western blot analysis of OSCC cells with pcDNA3.1(+) only or co-transfected with pmiR-198 and different *TOPORS* constructs. Note, a reduced level of TOPORS in SCC131 and SCC084 cells co-transfected with p*TOPORS*-3′UTR-S and pmiR-198 in comparison to those co-transfected with pmiR-198 and p*TOPORS* or pmiR-198 and p*TOPORS*-3′UTR-M, underscoring that miR-198 targets *TOPORS* by binding to its 3′UTR.

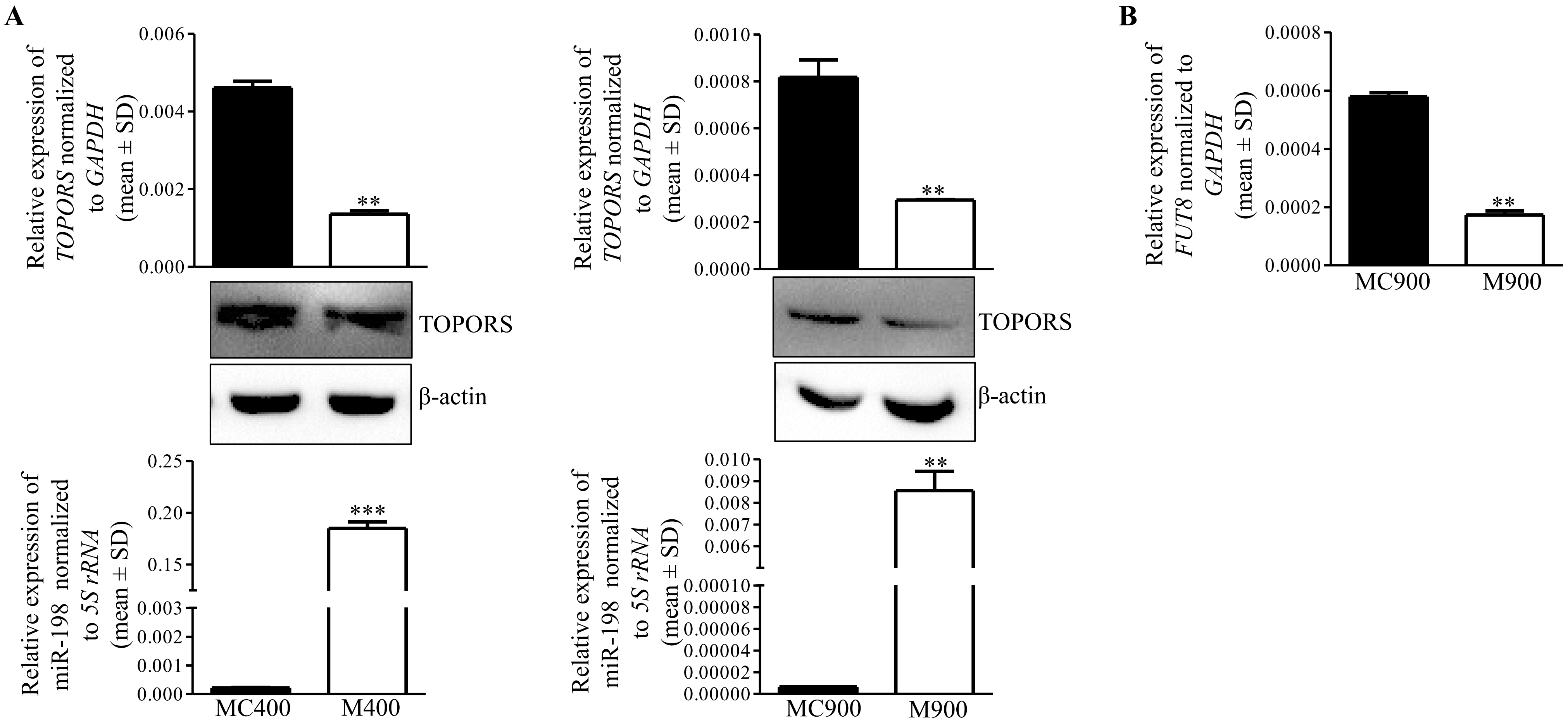

Supplementary Figure S4. Optimization of the dosage of a synthetic miR-198 mimic in SCC131 cells. **(A)** Levels of miR-198 and TOPORS in SCC131 cells transfection with 400 nM (M400) and 900 nM (M900) of a synthetic miR-198 mimic or a 400 nM (MC400) and 900 nM mimic Mock/control (MC900). Note, the dosage of 900 nM miR-198 mimic is optimum for cells as it shows a marked reduction of TOPORS at transcript and protein levels. **(B)** The level of *FUT8* (positive control) in cells transfected with 900 nM miR-198 mimic (M900) or mimic control (MC900). Each qRT-PCR data is an average of 2 technical replicates.

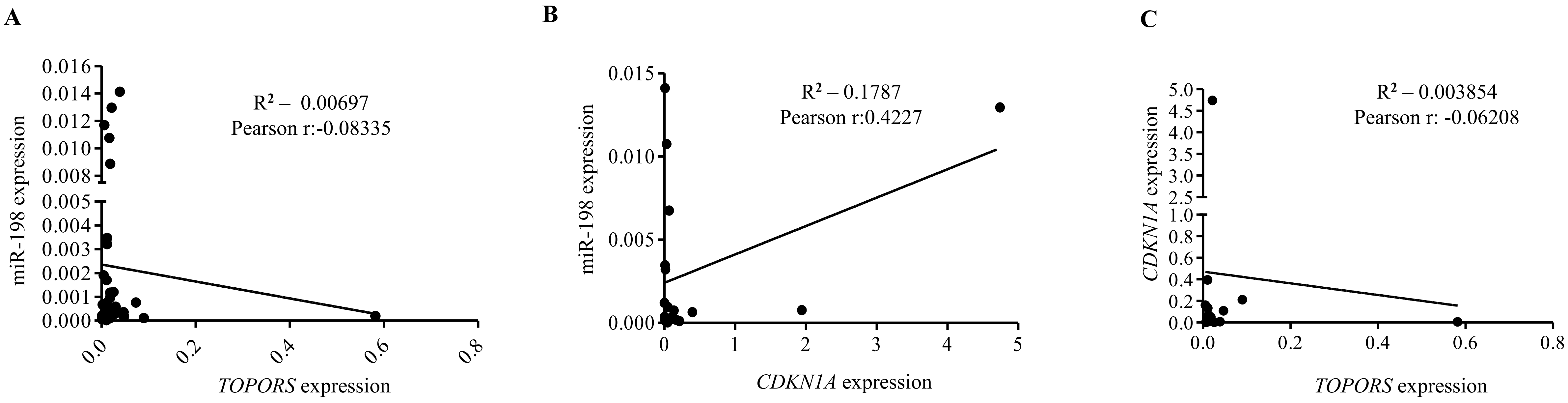

Supplementary Figure S5. Correlation studies. (A) Correlation of miR-198 and *TOPORS* expressions in patients. The p value is 0.6140. The negative r value indicates an inverse correlation between miR-198 and its target gene *TOPORS.* (**B)** Correlation of miR-198 and *CDKN1A* expressions in patients. The p value is 0.0806. The positive r value indicates a direct correlation between miR-198 and *CDKN1A* expressions. (**C)** Correlation of *TOPORS* and *CDKN1A* expressions in patients. The p value is 0.8067. The negative r value indicates an inverse correlation between *TOPORS* and *CDKN1A* expressions. The Pearson correlation analysis was performed to determine an association for the study using the Graphpad Prism 8. The p values are non-significant for all three sets. This is possibly due to a high variation among patient samples itself.

Supplementary Table ST1. A summary of the clinicopathological features of OSCC patients included in the study.

Characteristics	No. of patients (39)
**Median Age/Range (yr)**	55 (28-78)
**<50**	18 (46.15%)
**>51**	21 (53.84%)
**Gender**	
**Females**	20 (51.28%)
**Males**	19 (48.71%)
**Site of cancer**	
**BM**	10 (25.64%)
**Tongue**	5 (12.82%)
**GBS**	5 (12.82%)
**RMT**	2 (5.12%)
**Alveolus**	2 (5.12%)
**BM + GBS**	7 (17.94%)
**GBS + RMT**	3 (7.69%)
**GBS + Maxilla**	1 (2.56%)
**BM + GBS + RMT**	3 (7.69%)
**GBS + Maxilla**	1 (2.56%)
**Tumor grade**	
**Grade I**	25 (64.10%)
**Grade II**	13 (33.33%)
**Grade III**	1 (2.56%)
**Tumor classification**	
**T1**	0 (0%)
**T2**	3 (7.69%)
**T3**	16 (41.02%)
**T4**	20 (51.28%)
**Differentiation**	
**Well**	21 (53.84%)
**Moderate**	14 (35.89%)
**Poor**	4 (10.25%)

Abbreviations: yr, years; BM, buccal mucosa; GBS, gingiva-buccal sulcus; RMT, retromolar trigone; and T, tumor.

Supplementary Table ST2. Details of clinicopathological parameters of the patients included in the study.

Sl. No.	Pt. No.	Sex	Age (yr)	Tumor Grade	Cancer site	Differentiation	TNM	Habit
1	3	F	56	II	BM	Well	T3N2M1	Betel nut + Tob chewing
2	6	F	76	I	Alveolus	Well	T4aN1M0	Tob chewing
3	8	M	53	I	Tongue	Well	T3aN1M1	Paan + Tob chewing
4	10	M	31	I	BM	Well	T4aN1M0	Tob chewing
5	14	F	60	I	BM	Well	T4aN1M0	Tob chewing
6	15	F	42	I	BM+GBS	Well	T4aN1M1	Betel nut chewing
7	17	F	58	II	BM	Well	T4aN2M1	Betel nut chewing
8	24	M	45	II	BM+GBS+RMT	Well	T4N1M1	Areca nut +Tob chewing
9	25	M	52	I	BM+GBS+RMT	Well	T3aN1M0	Betel nut + Tob chewing
10	32	F	60	I	BM+GBS	Well	T4aN1M0	Tob chewing
11	33	F	45	I	GBS	Moderate	T3aN1M0	Betel nut chewing
12	43	F	39	I	RMT	Well	T4N1M0	Betel nut +Tob chewing
13	44	F	58	I	RMT	Well	T4aN1M0	Betel nut chewing
14	45	F	65	II	GBS+ Maxilla	Moderate	T4N2M0	Betel nut +Tob chewing
15	46	M	39	II	GBS+RMT	Poor	T3aN2M0	Tob chewing
16	47	M	54	I	GBS+BM+ Maxilla	Poor	T3aN1M0	Tob chewing
17	48	M	65	II	GBS+RMT	Well	T4N2M0	Paan Masala
18	49	F	78	II	GBS	Moderate	T3N2M0	Betel nut chewing
19	50	M	33	II	Tongue	Well	T4N2M0	Paan masala
20	51	F	41	I	BM	Moderate	T4aN1M0	Betel nut +Tob chewing
21	52	F	73	I	BM	Moderate	T3aN1M0	Betel nut chewing
22	53	F	65	I	BM+GBS	Moderate	T3aN1M0	Betel nut +Tob chewing
23	54	F	65	II	BM+GBS	Poor	T2N2M1	Tob chewing
24	55	F	70	I	BM+GBS	Well	T3aN1M0	No habit
25	56	F	69	II	GBS	Moderate	T3aN2M0	Betel nut + Tob chewing
26	57	M	72	I	Alveolus	Well	T4aN1M0	Tob chewing
27	59	M	31	I	GBS+RMT	Well	T4aN1M0	Tob chewing
28	60	F	49	III	BM+GBS	Moderate	T4aN2M1	Betel nut chewing
29	61	M	60	I	BM	Poor	T4aN2M0	Betel nut +Tob chewing
30	62	M	53	I	Tongue	Moderate	T3aN1M0	No habit
31	63	F	52	I	BM	Moderate	T2N1M0	Betel nut chewing
32	64	M	45	II	BM+GBS+RMT	Well	T3aN2M0	Paan chewing
33	65	M	44	I	Tongue	Well	T4aN1M0	Paan + Tob chewing
34	66	M	40	II	BM	Well	T4aN2M0	Tob chewing
35	67	F	43	II	BM+GBS	Well	T4aN2M0	Paan chewing
36	68	M	28	I	Tongue	Moderate	T2N1M0	Gutkha + Tob chewing +Cigarette
37	70	M	32	I	GBS	Moderate	T3aN1M0	Tob chewing
38	76	M	33	I	GBS	Moderate	T3aN1M0	Tob chewing
39	80	M	32	I	BM	Moderate	T3aN1M0	Tob chewing

Abbreviations: Pt. No., Patient number; yr, years; M, Male; F, Female; TNM, Tumor Node and Metastasis; BM, buccal mucosa; GBS, gingivo-buccal sulcus; RMT, retromolar trigone; and Tob, Tobacco chewing.

Supplementary Table ST3. Details of primers used in qRT-PCR.

Gene	Sequence (5' to 3')	Amplicon size (bp)	Annealing temp. (^oC)	Reference
GAPDH	FP: GAAGGGTGAAGGTCGGAGTC<br>RP: GAAGATGGTGATGGGATTTC	226	60	_
MIR198	RT6-miR-198:<br>TGTCAGGCAACCGTATTCACCGTGAGTGGTGAACCT<br>Short-miR-198:<br>CGTCAGATGTCCGAGTAGAGGGGGAACGGCGGGTCCAGAGGGGAGATAGG<br>MP- F: TGTCAGGCAACCGTATTCACC<br>MP- R: CGTCAGATGTCCGAGTAGAGG	86	60	(30);<br>Present study
5S rRNA	FP: GCCCGATCTCGTCTGATCT<br>RP: AGCCTACAGCACCCGGTATT	93	60	_
MCPH1	FP: TCACCACAGCGCAATGGAGAAGAGA<br>RP: ATCACGTGAAATGTTCAAAGGTGCTTC	145	62	(54)
TOPORS	FP: CGTGCGACGACTTACTGGCTTC<br>RP: TAGGTCGGTGTCGGCAGGATC	155	67	_
FUT8	FP: CGAATCTCTCCGGATACCAGAAG<br>RP: ACCAGAGCTCTTTAGCTCCATTTTC	191	53	_
TP53	FP: CTTGCCACAGGTCTCCCCAA<br>RP: AGGGGTCAGAGGCAAGCAGA	237	60	_
CDKN1A(P21)	FP: CCTCAAATCGTCCAGCGAC<br>RP: CATTGTGGGAGGAGCTGTG	393	55	_

Abbreviations: FP, forward primer; RP, reverse primer; bp, base pair; and, temp., temperature.

Supplementary Table ST4. Details of the plasmid constructs generated in the present study.

Construct	Cloning vector	Primer sequence (5' to 3')	Amplicon size (bp)	Annealing temp. (°C)
pmiR-198	pcDNA3-*EGFP*	FP: TAGCAAGCTTCCAGCCTACCAATATGCTCATTCGAC<br>*Hind* III<br>RP: AGCTCTCGAGTGAGGACTCCATATCATAGCACGACC<br>*Xho* I	311	52
pBSKS-*TOPORS*-3'UTR-S	pBSKS (+)	FP: ACTCGATATCATGAACGCGTAAGGAAGAATGTCGTCTACTGCA<br>*Eco* RV* *Mlu* I<br>RP: ATGGGCGGCCGCATGAGTTTAAACAGCAAGTTAAATGCTGTTTTTACTG<br>*Not* I * *Pme* I	769	56
pMIR-REPORT-*FUT8*-3'UTR-S (Positive control)	pMIR-REPORT™	FP: AAGTGAGCTCGCTGCAATGCCCTCATACCCATG<br>*Sac* I<br>RP: ATTGACGCGTAGCAGGGCATGATAGGCTAGGG<br>*Mlu* I	980	60
pMIR198-prom	pGL3-Basic	FP: AGCTCTCGAGCTGGTGCAGGGGCTGAAATAACC<br>*Xho* I<br>RP: TAGCAAGCTTCCAAATGCATCTGAAACAGGGTAG<br>*Hind* III	839	58
p*TOPORS*	pcDNA3.1(+)	Construct ordered from GenScript<br>The *TOPORS* ORF can be released by a double digestion with *Eco* RI and *Eco* RV	3138	–

Supplementary Table ST4. Details of the plasmid constructs generated in the present study (continued).

Plasmid constructs generated by site-directed mutagenesis				
**Construct**	**Template vector**	**Primer sequence (5' to 3')**	**Amplicon size (bp)**	**Annealing temp. (ºC)**
pMIR-REPORT-*TOPORS*-3'UTR-M	pBSKS-*TOPORS*-3'UTR-S	FP: TTATATACTTGTTTATGCCAGTAAAACAAA<br>RP: TTTGTTTTACTGGCATAAACAAGTATATAA	–	–
**Plasmid constructs generated by sub-cloning**				
pMIR-REPORT-*TOPORS*-3'UTR-S	pMIR-REPORT™	FP: ACTCGATATCATGAACGCGTAAGGAAGAATGTCGTCTACTGCA<br>*Eco* RV *Mlu* I*<br>RP: ATGGGCGGCCGCATGAGTTTAAACAGCAAGTTAAATGCTGTTTTTACTG<br>*Not* I *Pme* I*	-	-
p*TOPORS*-3'UTR-S	p*TOPORS*	FP: ACTCGATATCATGAACGCGTAAGGAAGAATGTCGTCTACTGCA<br>*Eco* RV * *Mlu* I<br>RP: ATGGGCGGCCGCATGAGTTTAAACAGCAAGTTAAATGCTGTTTTTACTG<br>*Not* I* *Pme* I	-	-
p*TOPORS*-3'UTR-M	p*TOPORS*	FP: ACTCGATATCATGAACGCGTAAGGAAGAATGTCGTCTACTGCA<br>*Eco* RV * *Mlu* I<br>RP: ATGGGCGGCCGCATGAGTTTAAACAGCAAGTTAAATGCTGTTTTTACTG<br>*Not* I* *Pme* I	-	-

Abbreviations: FP, forward primer; RP, reverse primer; bp, base pair; and, temp., temperature.
* Restriction enzymes used for cloning.

Supplementary Table ST5. Details of the putative *MIR198* promoter cloned in the pGL3-Basic vector.

**Construct**	**Database used for promoter prediction**	**Promoter fragment position w.r.t. TSS**	**Fragment length (bp)**
pMIR198-prom	DBTSS/MatInspector/Switchgear genomics	-407 to +432	839

Abbreviations: +1- TSS, Transcription Start Site; and bp, base pair.

Supplementary Table ST6. Details of primers used for bisulphite sequencing PCR analysis.

Primer Sequence (5' to 3')	Amplicon size (bp)	Annealing temp. (^oC)
miR-198-Meth FP: TGAGAAGTTGAGGTAAATTAG<br>miR-198-Meth RP: CCACTCTCTTCCTACTTTAC	403	56

Abbreviations: FP, forward primer; RP, reverse primer; bp, base pair; and, temp., temperature.

Supplementary Table ST7. A list of predicted gene targets* for miR-198.

**Target prediction programs**			
**miRDB**	**TargetScanHuman**	**PicTar**	**CoMeTa**
NRIP1	*HMGA1*	***PDCD1LG2***	***PUM2***
PUM2	***PDCD1LG2***	***SLC2A1***	***SLC2A1***
PBX1	***FUT8***	*PTEN*	***PBX1***
HMGA1	***PBX1***	*VCP*	***MET***
SCG2	***VCP***	***PUM2***	*HMGA1*
FUT8	***PUM2***	***NRIP1***	***VCP***
TOPORS*	***NRIP1***	***TOPORS****	*PLAU*
PLAU	*PLAU*	*MET*	***NRIP1***
MET	***TOPORS****	***PBX1***	***FUT8***
PDCD1LG2	*SCG2*	***FUT8***	***PDCD1LG2***
SLC2A1	***SLC2A1***	*OTX2*	***TOPORS****
VCP	***MET***	*ESRRA*	*SCG2*

*Gene targets highlighted in bold are predicted by all the four target prediction programs, of which *TOPORS* was further investigated in the present study.
